# Author Correction: Intratumoural immune heterogeneity as a hallmark of tumour evolution and progression in hepatocellular carcinoma

**DOI:** 10.1038/s41467-021-21556-y

**Published:** 2021-02-23

**Authors:** Phuong H. D. Nguyen, Siming Ma, Cheryl Z. J. Phua, Neslihan A. Kaya, Hannah L. H. Lai, Chun Jye Lim, Jia Qi Lim, Martin Wasser, Liyun Lai, Wai Leong Tam, Tony K. H. Lim, Wei Keat Wan, Tracy Loh, Wei Qiang Leow, Yin Huei Pang, Chung Yip Chan, Ser Yee Lee, Peng Chung Cheow, Han Chong Toh, Florent Ginhoux, Shridhar Iyer, Alfred W. C. Kow, Yock Young Dan, Alexander Chung, Glen K. Bonney, Brian K. P. Goh, Salvatore Albani, Pierce K. H. Chow, Weiwei Zhai, Valerie Chew

**Affiliations:** 1 Translational Immunology Institute (TII), SingHealth-DukeNUS Academic Medical Centre, Singapore, Singapore; 2 Genome Institute of Singapore (GIS), Agency for Science, Technology and Research (A*STAR), Singapore, Singapore; 3 School of Biological Sciences, Nanyang Technological University Singapore, Singapore, Singapore; 4 Cancer Science Institute of Singapore, National University of Singapore, Singapore, Singapore; 5 Department of Biochemistry, Yong Loo Lin School of Medicine, National University of Singapore, Singapore, Singapore; 6 Department of Pathology, Singapore General Hospital, Singapore, Singapore; 7 Duke-NUS Medical School, Singapore, Singapore; 8 Department of Pathology, National University Hospital Singapore, Singapore, Singapore; 9 National Cancer Centre, Singapore, Singapore; 10 Department of Hepatopancreatobiliary and Transplant Surgery, Singapore General Hospital, Singapore, Singapore; 11 Singapore Immunology Network (SIgN), A*STAR, Singapore, Singapore; 12 Division of Hepatobiliary & Pancreatic Surgery, Department of Surgery, University Surgical Cluster, National University Health System, Singapore, Singapore; 13 Department of Medicine, Yong Loo Lin School of Medicine, National University Hospital Singapore, Singapore, Singapore; 14 Key Laboratory of Zoological Systematics and Evolution, Institute of Zoology, Chinese Academy of Sciences, Beijing, China; 15 Center for Excellence in Animal Evolution and Genetics, Chinese Academy of Sciences, Kunming, China

**Keywords:** Cancer microenvironment, Hepatocellular carcinoma, Immunoediting

Correction to: *Nature Communications* 10.1038/s41467-020-20171-7, published online 11 January 2021.

The original version of this Article omitted from the author list the 25th author Glen K. Bonney, who is from ‘Division of Hepatobiliary & Pancreatic Surgery, Department of Surgery, University Surgical Cluster, National University Health System, Singapore’. Consequently, the following was added to the Author Contributions: ‘G.K.B recruited patients, provided samples and discussed the data. F.G. provided analysis support for immunomics data.’ The original version of this Article also contained an error in the author affiliations. Affiliation 12 incorrectly read ‘Department of Medicine, Yong Loo Lin School of Medicine, National University Hospital Singapore, Singapore, Singapore’. In addition, the original version of this Article contained an error in the author affiliations. Yock Young Dan was incorrectly associated with Division of Gastroenterology and Hepatology, National University Hospital Singapore, Singapore, Singapore. These errors have now been corrected in both the PDF and HTML versions of the Article.

