# Supplementary material for: Intratumoural immune heterogeneity as a hallmark of tumour evolution and progression in hepatocellular carcinoma
Source: Nat Commun. 2021 Jan 11;12:227. doi: 10.1038/s41467-020-20171-7 (PMC7801667; doi:10.1038/s41467-020-20171-7)
Supplement: Supplementary file 1 — Supplementary Information [file 41467_2020_20171_MOESM1_ESM.pdf]

Supplementary Tables and Figures

Supplementary Table 1: Clinical and demographic information of 28 HCC patients

Pat ID	Race	Stage (TNM)	Grade (Edmondson)	Viral status	Tumour size (cm)	No. of sectors	Tumour multiplicity	AFP level (ng/ml)	MVI	Fibrosis Stage	Recurrence
A001	Chinese	I	IV	NV	2.0	2	1	6.7	N	4	Y
A002	Chinese	I	II	Hep B	3.5	4	1	2.8	N	3	N
A004	Chinese	II	II	Hep B	3.0	2	1	38	Y	1	Y
A008	Malay	IB	II	NV	3.8	2	1	248	N	0	N
A009	Chinese	II	III	NV	7.5	4	1	2203	Y	0	N
B002	Chinese	I	II	Hep B	2.5	2	1	4.7	N	2	N
B003	Chinese	IIIC	III	NV	11.0	2	1	1.9	Y	3	Y
B006	Chinese	I	III	Hep B	3.6	3	1	8592	N	2	N
B008	Chinese	IIIC	III	Hep B	7.0	5	1	36.6	Y	3	Y
B009	Malay	IIIC	III	Hep B	9.5	5	1	>60500	Y	4	Y
B010	Chinese	I	I	NV	2.5	2	1	5.4	N	1	N
B012	Chinese	I	I	Hep B	2.6	2	1	4.2	N	4	N
B013	Chinese	IIIA	II	Hep B	8.0	4	multifocal satellite	49.9	N	2	Y
B014	Chinese	II	II	NV	14.0	4	1	1.5	Y	3	N
B015	Chinese	IB	II	Hep B	2.2	2	1	6.1	N	4	N
B016	Chinese	IB	II	Hep B	4.7	4	1	8920	N	0	N
B017	Chinese	II	III	Hep B	6.2	5	1	504	Y	2	Y
C002	Chinese	IIIA	II	NV	12.7	5	2	1069	N	3	Y
C003	Chinese	II	II	Hep B	3.5	3	2	3	N	4	N
C004	Chinese	I	II	Hep B	8.5	3	1	1021	N	2	Y
C005	Others	IIIB	III	NV	8.0	4	1	144	Y	4	Y
C008	Chinese	IIIA	II	Hep B	14.0	4	1	13.7	N	1	Y
C010	Indian	IB	II	NV	15.0	5	1	9.3	N	0	N
H247	Chinese	II	III	NV	3.5	3	1	27.3	Y	4	Y
H255	Chinese	II	III	Hep B	5.0	4	1	>10000	Y	4	Y
H264	Malay	II	II	NV	8.0	2	1	20.1	Y	2	Y
H276	Chinese	I	III	NV	5.5	3	1	3.2	N	3	N
H319	Indonesian	IIIC	III	NV	5.6	5	1	14483	Y	1	Y

Footnote:
Gender: 21 Males (75%); 7 Females (25%)
Age range: 47-82 years old
Stage TNM: Version 8
Viral Status: Hep B- Hepatitis B; NV - non-viral-related HCC (patients with no detectable HBV surface/core antigen)
AFP: Alpha-fetoprotein
MVI: microvascular invasion
N- No; Y- Yes;

Supplementary Table 2: Antibodies used for CyTOF staining

Antibodies	Clone	Vendor	Catalog number	Metal Isotopes	Working Concentration
CD45 (Barcode 1)	HI30	Fluidigm	#3089003B	89	5ug/ml
CD14	TüK4	Lifetechnologies	#Q10064	112/114	5ug/ml
CD45 (Barcode 2)	HI30	Biolegend	#304002	115	5ug/ml
HLA-DR	L243	Biolegend	#307602	139	6ug/ml
CD19	HIB19	Biolegend	#302202	141	5ug/ml
CD45RO	UCHL1	Biolegend	#304202	142	5ug/ml
CD3	UCHT1	Biolegend	#300402	143	5ug/ml
CD8	SK1	Biolegend	#344702	144	5ug/ml
T-bet	4B10	Biolegend	#644802	145	5ug/ml
TNFa	Mab11	Biolegend	#502902	146	3ug/ml
PD-1	EH12.2H7	Biolegend	#329902	147	7ug/ml
CD4	SK3	Biolegend	#344602	148	4ug/ml
VISTA	MAB71261	R&D Systems	#MAB71261	149	5ug/ml
CD103	B-Ly7	Ebioscience	#14-1038-82	150	6ug/ml
TIGIT	MBSA43	Ebioscience	#16-9500-85	151	6ug/ml
NKp46	9E2	Biolegend	#331902	152	5ug/ml
CD25	2A3	BD bioscience	#347640	153	5ug/ml
CD27	O323	Biolegend	#302802	154	5ug/ml
CTLA-4	BNI3	BD bioscience	#555850	155	5ug/ml
PD-L1	29E.2A3	Biolegend	#329719	156	5ug/ml
CD244	C1.7	Biolegend	#329502	157	6ug/ml
IL-10	JES3-9D7	Biolegend	#501402	158	4ug/ml
LAG-3	17B4	Abcam	#ab40466	159	5ug/ml
TIM-3	F38-2E2	Biolegend	#345002	160	5ug/ml
CCR7	G043H7	Biolegend	#353202	161	7ug/ml
CD56	NCAM16.2	BD bioscience	#559043	162	4ug/ml
CXCR3	G025H7	Biolegend	#353702	163	5ug/ml
GITR	621	Biolegend	#311602	164	6ug/ml
FoxP3	PCH101	Ebioscience	#14-4776-82	165	3ug/ml
Ki67	20Raj1	Ebioscience	#14-5699-82	166	3ug/ml
CD80	2D10	Biolegend	#305202	167	8ug/ml
IFN-y	B27	Biolegend	#506502	168	4ug/ml
IL-17A	BL168	Biolegend	#512302	169	3ug/ml
CCR6	G034E3	Biolegend	#353402	170	6ug/ml
CD45RA	JS-83	Ebioscience	#14-9979-82	171	3ug/ml
CD45 (Barcode 3)	HI30	Biolegend	#304002	172	5ug/ml
GranzymeB	CLB-GB11	Abcam	#ab103159	173	2ug/ml
CD137	4B4-1	Biolegend	#309802	174	4ug/ml
CCR5	T21/8	Biolegend	#321402	175	7ug/ml
CD69	FN50	Biolegend	#310902	176	5ug/ml
	Ir Intercalator	Fluidigm	#201192B	191/193	0.25uM
CD16	3G8	Fluidigm	#3209002B	209	5ug/ml

Supplementary Table 3: List of genomic regions with copy number variation frequency that are significantly associated with tumours with high immune-ITH

(Two-sided Fisher's Exact test and adjusted P-value by Benjamini-Hochberg corrected for multiple tests)

List of significantly top 50 deleted genomic regions in tumours with high immune-ITH

Cytoband	Adjusted p-value	Genes
4q35.1	6.83E-05	ODZ3, DCTD, FAM92A3, C4orf38, WWC2, CLDN22, CLDN24, CDKN2AIP, LOC389247, ING2, RWDD4, TRAPPC11, STOX2, ENPP6, LOC728175, IRF2, CASP3, CCDC111, MLF1IP, ACSL1, SLED1, LOC731424, MIR3945, LOC100506229, HELT, SLC25A4, KIAA1430, SNX25, LRP2BP, ANKRD37, UFSP2, C4orf47, CCDC110, PDLIM3, SORBS2, TLR3, FAM149A
4p15.31	6.83E-05	DCAF16, NCAPG, LCORL, SLIT2, SLIT2-IT1, MIR218-1, hsa-mir-218-1, PACRGL, KCNIP4
4p15.32	6.83E-05	C1QTNF7, CC2D2A, FBXL5, FAM200B, BST1, CD38, FGFBP1, FGFBP2, PROM1, TAPT1, FLJ39653, LDB2, QDPR, CLRN2, LAP3, MED28, FAM184B
4q13.2	6.83E-05	hsa-mir-1269, CENPC1, STAP1, UBA6, LOC550112, GNRHR, TMPRSS11D, TMPRSS11A, TMPRSS11GP, LOC550113, TMPRSS11F, SYT14L, FTLP10, TMPRSS11BNL, TMPRSS11B, YTHDC1, TMPRSS11E, UGT2B17, UGT2B15, UGT2B10, UGT2A3, UGT2B7, UGT2B11, UGT2B28, UGT2B4, UGT2A1, UGT2A2
4q13.3	6.83E-05	SULT1B1, SULT1E1, CSN1S1, CSN2, STATH, HTN3, HTN1, CSN1S2AP, CSN1S2BP, C4orf40, ODAM, FDCSP, CSN3, CABS1, SMR3A, SMR3B, PROL1, MUC7, AMBN, AMTN, ENAM, IGJ, UTP3, RUFY3, GRSF1, MOB1B, DCK, SLC4A4, GC, NPFFR2, ADAMTS3, COX18, ANKRD17, ALB, AFP, AFM, RASSF6, IL8, CXCL6, PF4V1, CXCL1, PF4, PPBP, CXCL5, CXCL3, PPBPL2, CXCL2, MTHFD2L, EPGN, EREG, AREG, BTC, PARM1, LOC441025
4p15.33	6.83E-05	MIR572, hsa-mir-572, HS3ST1, HSP90AB2P, RAB28, LOC285547, NKX3-2, LOC285548, BOD1L, LOC152742, LOC441009, CPEB2
11p14.1	0.000115	CCDC34, LGR4, LIN7C, BDNF-AS1, BDNF, KIF18A, MIR610, hsa-mir-610, METTL15, KCNA4, FSHB, C11orf46, MPPED2, DCDC5
11p14.2	0.000115	ANO3, MUC15, SLC5A12, FIBIN, BBOX1
11p13	0.000247	DCDC1, DNAJC24, IMMP1L, ELP4, PAX6, DKFZp686K1684, RCN1, WT1, WT1-AS, EIF3M, CCDC73, PRRG4, QSER1, DEPDC7, TCP11L1, LINC00294, CSTF3, LOC338739, HIPK3, C11orf41, C11orf91, CD59, FBXO3, LMO2, CAPRIN1, NAT10, ABTB2, CAT, ELF5, EHF, APIP, PDHX, MIR1343, CD44, SLC1A2, PAMR1, FJX1, TRIM44, LDLRAD3, MIR3973, COMMD9, PRR5L
11p15.4	0.000262	KCNQ1DN, CDKN1C, SLC22A18AS, SLC22A18, PHLDA2, NAP1L4, SNORA54, CARS, OSBPL5, C11orf36, MRGPRG, MRGPRE, ZNF195, LOC650368, OR7E12P, TRPC2, ART5, ART1, CHRNA10, NUP98, PGAP2, RHOG, MIR4687, STIM1, RRM1, OR52B4, TRIM21, OR52K2, OR52K1, OR52M1, C11orf40, OR52I2, OR52I1, TRIM68, OR51D1, OR51E1, OR51E2, OR51F1, OR52R1, OR51F2, OR51S1, OR51T1, OR51A7, OR51G2, OR51G1, OR51A4, OR51A2, MMP26, OR51L1, OR52J3, OR52E2, OR52A5, OR52A1, OR51V1, HBB, HBD, HBBP1, HBG1, HBG2, HBE1, OR51B4, OR51B2, OR51B5, OR51B6, OR51M1, OR51Q1, OR51I1, OR51I2, OR52D1, UBQLN3, UBQLNL, OR52H1, OR52B6, TRIM6-TRIM34, TRIM6, TRIM34, TRIM78P, TRIM5, TRIM22, OR56B1, OR52N4, OR52N5, OR52N1, OR52N2, OR52E6, OR52E8, OR52E4, OR56A3, OR56A5, OR52L1, OR56A4, OR56A1, OR56B4, OR52B2, OR52W1, C11orf42, FAM160A2, CNGA4, CCKBR, PRKCDBP, APBB1, SMPD1, HPX, TRIM3, ARFIP2, FXC1, DNHD1, ILK, RRP8, TAF10, TPP1, DCHS1, MRPL17, GVINP1, OR2AG2, OR2AG1, OR6A2, OR10A5, OR10A2, OR10A4, OR2D2, OR2D3, ZNF215, ZNF214, NLRP14, RBMXL2, hsa-mir-302e, SYT9, OLFML1, PPFIBP2, CYB5R2, OVCH2, OR5P2, OR5P3, LOC283299, OR5E1P, OR10A6, OR10A3, NLRP10, EIF3F, TUB, RIC3, LMO1, STK33, TRIM66, RPL27A, SNORA3, SNORA45, ST5, AKIP1, C11orf16, ASCL3, TMEM9B, NRIP3, SCUBE2, KRT8P41, DENND5A, TMEM41B, IPO7, SNORA23, LOC644656, ZNF143, WEE1, SWAP70, LOC440028, LOC283104, SBF2, ADM, AMPD3, MIR4485, MTRNR2L8, RNF141, MRVI1-AS1, LYVE1, MRVI1
11p15.5	0.000433	LOC100133161, LOC653486, SCGB1C1, BET1L, ODF3, RIC8A, SIRT3, PSMD13, NLRP6, ATHL1, IFITM5, IFITM2, IFITM1, IFITM3, B4GALNT4, PKP3, SIGIRR, ANO9, PTDSS2, RNH1, HRAS, LRRC56, C11orf35, RASSF7, LOC143666, MIR210HG, MIR210, hsa-mir-210, PHRF1, IRF7, CDHR5, SCT, DRD4, DEAF1, TMEM80, EPS8L2, TALDO1, PDDC1, NS3BP, CEND1, SLC25A22, PIDD, RPLP2, SNORA52, PNPLA2, EFCAB4A, CD151, POLR2L, TSPAN4, CHID1, AP2A2, MUC6, MUC2, MUC5B, TOLLIP, LOC255512, BRSK2, MOB2, DUSP8, LOC338651, KRTAP5-1, KRTAP5-2, KRTAP5-3, KRTAP5-4, KRTAP5-5, FAM99A, FAM99B, KRTAP5-6, IFITM10, CTSD, SYT8, TNNI2, LSP1, MIR4298, hsa-mir-4298, TNNT3, MRPL23, MRPL23-AS1, H19, MIR675, hsa-mir-675, IGF2, INS-IGF2, MIR483, hsa-mir-483, IGF2-AS1, INS, MIR4686, TH, ASCL2, C11orf21, TSPAN32, CD81, TRPM5, TSSC4, KCNQ1, KCNQ1OT1
11p14.3	0.000433	ANO5, SLC17A6, FANCF, GAS2, SVIP, LOC100500938, LUZP2
11p15.1	0.000433	C11orf58, PLEKHA7, OR7E14P, RPS13, PIK3C2A, NUCB2, B7H6, KCNJ11, ABCC8, USH1C, MYOD1, KCNC1, SERGEF, TPH1, SAAL1, SAA3P, MRGPRX3, MRGPRX4, LOC494141, SAA2-SAA4, SAA2, SAA4, SAA1, HPS5, GTF2H1, LDHA, LDHC, hsa-mir-3159, LDHAL6A, TSG101, UEVLD, LOC100506540, SPTY2D1, TMEM86A, IGSF22, PTPN5, MRGPRX1, MRGPRX2, ZDHHC13, CSRP3, E2F8, NAV2, NAV2-AS4, MIR4486, LOC100126784, MIR4694, DBX1, HTATIP2, PRMT3, SLC6A5, NELL1
11p15.2	0.000433	LOC100506305, RASSF10, ARNTL, BTBD10, PTH, FAR1, SPON1, RRAS2, COPB1, PSMA1, PDE3B, CYP2R1, CALCA, CALCB, INSC, SOX6
11p15.3	0.000433	CTR9, EIF4G2, SNORD97, ZBED5, LOC729013, GALNTL4, CSNK2A1P, MIR4299, hsa-mir-4299, USP47, DKK3, MICAL2, MICALCL, PARVA, TEAD1
4q13.1	0.000433	LPHN3, TECRL, LOC401134, EPHA5, LOC100144602
5q22.3	0.000454	KCNN2, TRIM36, PGGT1B, CCDC112, FEM1C, TICAM2, TMED7-TICAM2, TMED7, CDO1, ATG12, AP3S1
4q33	0.000687	NEK1, CLCN3, C4orf27, LOC100506085, MFAP3L, AADAT, HSP90AA6P
4q34.1	0.000687	LOC100506122, GALNTL6, GALNT7, hsa-mir-548t, HMGB2, SAP30, SCRG1, HAND2, NBLA00301, FBXO8, CEP44, MIR4276, hsa-mir-4276, HPGD, GLRA3, ADAM29
4q34.2	0.000687	GPM6A, WDR17, SPATA4, ASB5, SPCS3
5q23.2	0.000687	ZNF474, LOC100505841, SNCAIP, SNX2, SNX24, PPIC, PRDM6, CEP120, CSNK1G3, ZNF608, GRAMD3, ALDH7A1, PHAX, C5orf48, LMNB1, MARCH3, C5orf63, MEGF10, PRRC1, CTXN3
4p15.1	0.000687	MIR4275, hsa-mir-4275, PCDH7
4p15.2	0.000687	KCNIP4-IT1, LOC100505912, GPR125, GBA3, MIR548AJ2, PPARGC1A, MIR573, hsa-mir-573, DHX15, SOD3, CCDC149, LGI2, SEPSECS, LOC285540, PI4K2B, ZCCHC4, ANAPC4, SLC34A2, SEL1L3, C4orf52, RBPJ, CCKAR, TBC1D19, STIM2
5q22.2	0.000687	FLJ11235, APC, SRP19, REEP5, DCP2, MCC, TSSK1B, YTHDC2
5q23.1	0.000687	AQPEP, COMMD10, LOC644100, SEMA6A, LOC728342, DTWD2, MIR1244-1|chr5, MIR1244-2|chr5, MIR1244-3|chr5, hsa-mir-1244-2, DMXL1, TNFAIP8, HSD17B4, FAM170A, PRR16, FTMT, SRFBP1, LOX
4q21.23	0.000687	COQ2, HPSE, HELQ, MRPS18C, FAM175A, AGPAT9, NKX6-1, CDS1, WDFY3, WDFY3-AS2, ARHGAP24, MIR4451
4q21.3	0.000687	MAPK10, PTPN13, SLC10A6, C4orf36, LOC100506746, AFF1
9p21.3	0.000687	MLLT3, MIR4473, MIR4474, KIAA1797, MIR491, hsa-mir-491, PTPLAD2, IFNB1, IFNW1, IFNA21, IFNA4, IFNA7, IFNA10, IFNA16, IFNA17, IFNA14, IFNA22P, IFNA5, KLHL9, IFNA13, IFNA1, IFNA2, IFNA6, IFNA8, IFNE, MIR31HG, MIR31, hsa-mir-31, MTAP, C9orf53, CDKN2A, CDKN2B-AS1, CDKN2B, DMRTA1, FLJ35282, ELAVL2
5q23.3	0.000687	FLJ33630, SLC12A2, FBN2, SLC27A6, ISOC1, MIR4633, MIR4460, ADAMTS19, CHSY3, HINT1, LYRM7, CDC42SE2
4q35.2	0.000687	CYP4V2, FLJ38576, KLKB1, F11, LOC285441, MTNR1A, FAT1, LOC339975, ZFP42, TRIML2, TRIML1, LOC401164, HSP90AA4P, FRG1, FRG2, LOC100288255, DUX2|chr4, DUX4L2|chr4, DUX4L3|chr4, DUX4L4, DUX4L5|chr4, DUX4L6|chr4, DUX4|chr4

List of significantly top 50 deleted genomic regions (cont.)

Cytoband	Adjusted p-value	Genes
4p16.2	0.000754	LOC100507266, MSX1, CYTL1, STK32B, C4orf6, EVC2, EVC, CRMP1, MIR378D1
4p16.3	0.000754	ZNF595, ZNF718, ZNF876P, ZNF732, ZNF141, hsa-mir-571, ABCA11P, ZNF721, PIGG, PDE6B, ATP5I, MYL5, MFSD7, PCGF3, CPLX1, LOC100129917, GAK, TMEM175, DGKQ, SLC26A1, IDUA, FGFRL1, RNF212, TMED11P, SPON2, LOC100130872, CTBP1, C4orf42, MAEA, KIAA1530, CRIPAK, FAM53A, SLBP, TMEM129, TACC3, FGFR3, LETM1, WHSC1, SCARNA22, WHSC2, MIR943, hsa-mir-943, C4orf48, NAT8L, POLN, HAUS3, MIR4800, MXD4, ZFYVE28, LOC402160, RNF4, FAM193A, TNIP2, ADD1, SH3BP2, MFSD10, C4orf10, NOP14, GRK4, HTT-AS1, HTT, C4orf44, RGS12, HGFAC, DOK7, LRPAP1, FLJ35424, LOC100133461, ADRA2C, FAM86EP, OTOP1, TMEM128, LYAR, ZBTB49, D4S234E, STX18
4q12	0.000754	DCUN1D4, LRRC66, SGCB, SPATA18, USP46, DANCR, MIR4449, SNORA26, ERVMER34-1, LOC152578, RASL11B, SCFD2, FIP1L1, LNX1, RPL21P44, CHIC2, GSX2, PDGFRA, KIT, KDR, SRD5A3, LOC100506462, TMEM165, CLOCK, PDCL2, NMU, LOC644145, EXOC1, CEP135, KIAA1211, AASDH, PPAT, PAICS, SRP72, ARL9, LOC100506564, HOPX, SPINK2, REST, NOA1, POLR2B, IGFBP7, LOC255130
4q21.1	0.001057	RCHY1, THAP6, C4orf26, CDKL2, G3BP2, USO1, PPEF2, NAAA, SDAD1, CXCL9, ART3, CXCL10, CXCL11, NUP54, SCARB2, FAM47E, FAM47E-STBD1, STBD1, CCDC158, SHROOM3, MIR4450, SOWAHB, SEPT11, CCNI, CCNG2, CXCL13, CNOT6L, MRPL1
4q21.21	0.001057	FRAS1, ANXA3, LOC100505702, BMP2K, PAQR3, LOC100505875, NAA11, GK2, LOC100506035, GDEP, ANTXR2, PRDM8, FGF5, C4orf22, BMP3, PRKG2, RASGEF1B
4q21.22	0.001057	HNRNPD, HNRPDL, ENOPH1, TMEM150C, C4orf11, SCD5, MIR575, hsa-mir-575, SEC31A, LOC100499177, THAP9, LIN54, COPS4, PLAC8
5q13.3	0.001057	ENC1, HEXB, GFM2, NSA2, FAM169A, GCNT4, ANKRD31, HMGCR, COL4A3BP, POLK, POC5, SV2C, IQGAP2, F2RL2, F2R, NCRUPAR, F2RL1, S100Z, CRHBP, AGGF1, SNORA47, ZBED3, LOC728723, PDE8B, WDR41
11q23.1	0.001057	ARHGAP20, C11orf53, C11orf92, C11orf93, MIR4491, POU2AF1, LOC100132078, BTG4, MIR34B, MIR34C, hsa-mir-34b, hsa-mir-34c, C11orf88, LAYN, SIK2, PPP2R1B, ALG9, FDXACB1, C11orf1, CRYAB, HSPB2-C11orf52, HSPB2, C11orf52, DIXDC1, DLAT, PIH1D2, C11orf57, TIMM8B, SDHD, IL18, TEX12, BCO2, PTS, C11orf34
5q14.1	0.001057	OTP, TBCA, AP3B1, SCAMP1, LHFPL2, ARSB, DMGDH, BHMT2, BHMT, JMY, HOMER1, PAPD4, CMYA5, MTX3, THBS4, SERINC5, LOC644936, SPZ1, CRSP8P, ZFYVE16, FAM151B, ANKRD34B, DHFR, MSH3, MTRNR2L2, RASGRF2, RNU5D-1, RNU5E-1, CKMT2, LOC100131067, ZCCHC9, ACOT12, SSBP2, ATG10
5q14.2	0.001057	RPS23, ATP6AP1L, MIR3977, TMEM167A, SCARNA18, XRCC4, VCAN
5q14.3	0.001057	HAPLN1, EDIL3, NBPF22P, COX7C, MIR3607, MIR4280, hsa-mir-4280, RASA1, CCNH, TMEM161B, LOC100505894, LINC00461, MIR9-2, hsa-mir-9-2, MEF2C, MIR3660, CETN3, MBLAC2, POLR3G, LYSMD3, GPR98, ARRDC3, LOC100129716
5q15	0.001057	FLJ42709, NR2F1, FAM172A, MIR2277, hsa-mir-2277, POU5F2, KIAA0825, ANKRD32, MCTP1, FAM81B, TTC37, ARSK, GPR150, RFESD, SPATA9, RHOBTB3, GLRX, C5orf27, ELL2, MIR583, hsa-mir-583, PCSK1, CAST, ERAP1, ERAP2, LNPEP, LIX1, RIOK2, FLJ35946, RGMB, CHD1
5q21.1	0.001057	LOC100289230, LOC100133050, FAM174A, ST8SIA4, hsa-mir-548p, SLCO4C1, SLCO6A1, PAM, GIN1, PPIP5K2, C5orf30
5q21.2	0.001057	NUDT12, RAB9BP1
5q21.3	0.001057	EFNA5, FBXL17, FER, PJA2, MAN2A1, LOC100289673
11q22.3	0.001068	DCUN1D5, DYNC2H1, MIR4693, PDGFD, DDI1, CASP12, CASP4, LOC643733, CASP5, CARD16, CASP1, CARD17, CARD18, GRIA4, KIAA1826, KBTBD3, AASDHPPT, GUCY1A2, CWF19L2, ALKBH8, ELMOD1, LOC643923, SLN, SLC35F2, RAB39A, CUL5, ACAT1, NPAT, ATM, C11orf65, KDELC2, EXPH5, DDX10, C11orf87, ZC3H12C, RDX, FDX1
11q23.2	0.001068	NCAM1, LOC100288346, TTC12, ANKK1, DRD2, MIR4301, hsa-mir-4301, TMPRSS5, ZW10, CLDN25, USP28, HTR3B, HTR3A, ZBTB16, NNMT, C11orf71, RBM7, REXO2, FAM55A, FAM55D
15q13.3	0.001068	MTMR10, TRPM1, MIR211, hsa-mir-211, LOC283710, KLF13, OTUD7A, CHRNA7, LOC100288615, ARHGAP11A, SCG5, GREM1, FMN1, TMCO5B
15q14	0.001068	RYR3, AVEN, CHRM5, C15orf24, PGBD4, C15orf29, TMEM85, SLC12A6, C15orf55, NOP10, LPCAT4, GOLGA8A, MIR1233-1, MIR1233-2, hsa-mir-1233-1, GOLGA8B, hsa-mir-1233-2, GJD2, ACTC1, AQR, ZNF770, ANP32AP1, ATPBD4, MIR3942, LOC100507466, MIR4510, C15orf41, CSNK1A1P1, LOC145845, MEIS2, TMCO5A, SPRED1, FAM98B, RASGRP1, C15orf53, C15orf54, THBS1, FSIP1, GPR176
15q15.1	0.001068	EIF2AK4, LOC100131089, SRP14, BMF, BUB1B, PAK6, C15orf56, ANKRD63, PLCB2, C15orf52, PHGR1, DISP2, C15orf23, IVD, BAHD1, CHST14, MRPL42P5, C15orf57, RPUSD2, CASC5, LOC100505648, RAD51, FAM82A2, C15orf62, DNAJC17, GCHFR, ZFYVE19, PPP1R14D, SPINT1, RHOV, VPS18, DLL4, CHAC1, INO80, EXD1, CHP, OIP5-AS1, OIP5, NUSAP1, NDUFAF1, RTF1, ITPKA, LTK, RPAP1, TYRO3, MGA, MIR626, hsa-mir-626, MAPKBP1, JMJD7-PLA2G4B, JMJD7, PLA2G4B, SPTBN5, MIR4310, hsa-mir-4310, EHD4, PLA2G4E, PLA2G4D, PLA2G4F, VPS39, MIR627, hsa-mir-627, TMEM87A, GANC, CAPN3, ZFP106, SNAP23

List of top 50 significantly amplified genomic regions in tumours with high immune-ITH

Cytoband	Adjusted p-value	Genes
1q21.2	0.000711	BCL9, ACP6, GJA5, GJA8, GPR89B, NBPF14, PPIAL4D, PPIAL4F, NBPF15, NBPF16, PPIAL4E, LOC645166, LOC388692, FCGR1C, HIST2H2BF, FAM91A2, BOLA1, FCGR1A, HIST2H2AA3, HIST2H2AA4, HIST2H2AB, HIST2H2AC, HIST2H2BC, HIST2H2BE, HIST2H3A, HIST2H3C, HIST2H3D, HIST2H4A, HIST2H4B, MTMR11, OTUD7B, SF3B4, SV2A, VPS45, PLEKHO1, ANP32E, CA14, APH1A, C1orf54, C1orf51, MRPS21, PRPF3
1q21.3	0.000711	RPRD2, TARS2, ECM1, ADAMTSL4, MIR4257, hsa-mir-4257, MCL1, ENSA, GOLPH3L, HORMAD1, CTSS, CTSK, ARNT, SETDB1, CERS2, ANXA9, FAM63A, PRUNE, BNIPL, C1orf56, CDC42SE1, MLLT11, GABPB2, SEMA6C, LYSMD1, SCNM1, TMOD4, TNFAIP8L2-SCNM1, TNFAIP8L2, VPS72, PIP5K1A, PSMD4, ZNF687, PI4KB, RFX5, SELENBP1, POGZ, PSMB4, CGN, TUFT1, MIR554, hsa-mir-554, SNX27, CELF3, RIIAD1, MRPL9, OAZ3, TDRKH, LINGO4, RORC, C2CD4D, LOC100132111, THEM5, THEM4, S100A10, S100A11, TCHHL1, TCHH, RPTN, HRNR, FLG, FLG2, CRNN, CRCT1, LCE5A, LCE3E, LCE3D, LCE3C, LCE3B, LCE3A, LCE2D, LCE2C, LCE2B, LCE2A, LCE4A, C1orf68, KPRP, LCE1F, LCE1E, LCE1D, LCE1C, LCE1B, LCE1A, LCE6A, SMCP, IVL, SPRR4, SPRR1A, SPRR3, SPRR1B, SPRR2D, SPRR2A, SPRR2B, SPRR2E, SPRR2F, SPRR2C, SPRR2G, LELP1, PRR9, LOR, PGLYRP3, PGLYRP4, S100A9, S100A12, S100A8, S100A7A, S100A7L2, S100A7, S100A2, S100A3, S100A4, S100A5, S100A6, S100A16, S100A13, S100A14, S100A1, CHTOP, SNAPIN, ILF2, NPR1, INTS3, SLC27A3, GATAD2B, DENND4B, CRTC2, SLC39A1, CREB3L4, JTB, RAB13, NUP210L, RPS27, TPM3, MIR190B, hsa-mir-190b, C1orf189, C1orf43, UBAP2L, HAX1, AQP10, ATP8B2, IL6R, SHE, TDRD10, UBE2Q1, CHRNB2, ADAR, KCNN3, PMVK, PBXIP1, PYGO2, SHC1, CKS1B, MIR4258, hsa-mir-4258, FLAD1, LENEP, ZBTB7B, DCST2
17q23.2	0.001154	SCARNA20, C17orf64, APPBP2, PPM1D, BCAS3, TBX2, C17orf82, TBX4, NACA2, BRIP1, INTS2, MED13, TBC1D3P2, EFCAB3, METTL2A, TLK2, MRC2, MARCH10, MIR548W, hsa-mir-633, TANC2
17q23.3	0.001154	CYB561, ACE, KCNH6, DCAF7, TACO1, MAP3K3, LIMD2, LOC729683, STRADA, CCDC47, DDX42, FTSJ3, PSMC5, SMARCD2, TCAM1P, CSH2, GH2, CSH1, CSHL1, GH1, CD79B, SCN4A, C17orf72, ICAM2, ERN1, SNORA76, SNORD104, TEX2, PECAM1, MILR1, POLG2, DDX5, MIR3064, MIR5047, CEP95, SMURF2
17q24.1	0.001154	LOC146880, PLEKHM1P, hsa-mir-4315-2, LRRC37A3, AMZ2P1, GNA13, RGS9, AXIN2, CEP112
17q24.2	0.001154	APOH, PRKCA, MIR634, hsa-mir-634, CACNG5, CACNG4, CACNG1, HELZ, PSMD12, PITPNC1, hsa-mir-548d-2, NOL11, SNORA38B, BPTF, C17orf58, KPNA2, LOC100499466, LOC440461, AMZ2, ARSG, SLC16A6, WIPI1, MIR635, hsa-mir-635, PRKAR1A, FAM20A, ABCA8, ABCA9, ABCA6, MIR4524A
17q24.3	0.001154	ABCA10, ABCA5, MAP2K6, KCNJ16, KCNJ2-AS1, KCNJ2, SOX9, LOC100499467, LINC00511, SLC39A11
1q21.1	0.001154	hsa-mir-3118-1, LOC375010, hsa-mir-3118-2, hsa-mir-3118-3, FLJ39739, LOC100130000, LOC100286793, PPIAL4G, FAM72D, SRGAP2P2, LOC728855, LOC728875, PPIAL4A, PPIAL4B, PPIAL4C, PFN1P2, NBPF9, LOC653513, PDE4DIP, SEC22B, NOTCH2NL, NBPF10, HFE2, TXNIP, POLR3GL, ANKRD34A, LIX1L, GNRHR2, RBM8A, PEX11B, ITGA10, ANKRD35, PIAS3, NUDT17, POLR3C, RNF115, CD160, PDZK1, GPR89A, GPR89C, PDZK1P1, NBPF11, NBPF24, LOC728989, PRKAB2, PDIA3P, FMO5, CHD1L, LOC100289211
1q22	0.001154	ADAM15, DCST1, EFNA4, LOC100505666, EFNA3, EFNA1, DPM3, SLC50A1, KRTCAP2, TRIM46, MUC1, MIR92B, THBS3, hsa-mir-92b, MTX1, GBAP1, GBA, FAM189B, SCAMP3, CLK2, HCN3, PKLR, FDPS, RUSC1-AS1, RUSC1, ASH1L, MIR555, hsa-mir-555, POU5F1P4, LOC645676, MSTO1, YY1AP1, DAP3, MSTO2P, GON4L, SYT11, RIT1, KIAA0907, SNORA42, SCARNA4, ARHGEF2, RXFP4, SSR2, UBQLN4, LAMTOR2, RAB25, MEX3A, LMNA, SEMA4A, SLC25A44, PMF1-BGLAP, PMF1, BGLAP, PAQR6, SMG5, TMEM79, C1orf85, VHLL, CCT3, C1orf182, RHBG, C1orf61, MIR9-1, hsa-mir-9-1, MEF2D, IQGAP3
6p11.2	0.001154	BAG2, RAB23, PRIM2, hsa-mir-548u, GUSBP4
14q22.2	0.001154	BMP4, CDKN3, CNIH, GMFB, CGRRF1, SAMD4A, GCH1, MIR4308, hsa-mir-4308, WDHD1, SOCS4
14q22.3	0.001154	MAPK1IP1L, LGALS3, DLGAP5, FBXO34, ATG14, TBPL2, KTN1-AS1, KTN1, RPL13AP3, LINC00520, PELI2, C14orf101, OTX2, OTX2OS1, EXOC5, MUDENG, NAA30, C14orf105, SLC35F4
14q23.1	0.001154	C14orf37, ACTR10, PSMA3, FLJ31306, ARID4A, TOMM20L, TIMM9, KIAA0586, DACT1, DAAM1, GPR135, C14orf149, JKAMP, C14orf38, RTN1, C14orf135, DHRS7, PPM1A, C14orf39, SIX6, SIX1, SIX4, MNAT1, TRMT5, SLC38A6, TMEM30B, PRKCH, FLJ22447
6p21.31	0.001508	BAK1, GGNBP1, ITPR3, LINC00336, MNF1, IP6K3, LEMD2, MLN, GRM4, LOC100507584, MIR1275, hsa-mir-1275, HMGA1, C6orf1, NUDT3, RPS10-NUDT3, RPS10, PACSIN1, SPDEF, C6orf106, SNRPC, UHRF1BP1, TAF11, ANKS1A, TCP11, SCUBE3, ZNF76, DEF6, PPARD, FANCE, RPL10A, TEAD3, TULP1, FKBP5, LOC285847, ARMC12, CLPSL2, CLPSL1, CLPS, LHFPL5, SRPK1, SLC26A8, MAPK14, MAPK13, BRPF3, PNPLA1, C6orf222, ETV7, PXT1, KCTD20, STK38, SRSF3, MIR3925
6p22.1	0.001569	HIST1H2AG, HIST1H2AH, HIST1H2BJ, HIST1H2BK, HIST1H4I, MIR3143, PRSS16, hsa-mir-3143, POM121L2, VN1R10P, ZNF204P, ZNF391, ZNF184, LOC100507173, LOC100131289, HIST1H2AI, HIST1H2BL, HIST1H2AJ, HIST1H2BM, HIST1H3H, HIST1H4J, HIST1H2AK, HIST1H2BN, HIST1H4K, HIST1H1B, HIST1H2AL, HIST1H3I, HIST1H4L, HIST1H2AM, HIST1H2BO, HIST1H3J, OR2B2, OR2B6, ZNF165, ZSCAN12P1, ZSCAN16, ZNF192, TOB2P1, ZNF193, ZKSCAN4, NKAPL, ZNF187, PGBD1, ZNF323, ZKSCAN3, ZSCAN12, ZSCAN23, GPX6, GPX5, SCAND3, LOC401242, TRIM27, ZNF311, LOC100129636, OR2W1, OR2B3, OR2J3, OR2J2, OR14J1, OR5V1, OR12D3, OR12D2, OR11A1, OR10C1, OR2H1, MAS1L, LOC100507362, UBD, OR2H2, SNORD32B, GABBR1, MOG, ZFP57, HLA-F-AS1, HLA-F, IFITM4P, HCG4, LOC554223, HLA-G, HLA-H, HCG4B, HLA-A, HCG9, ZNRD1-AS1, HLA-J, PPP1R11, RNF39, ZNRD1, TRIM31, TRIM40, TRIM10, TRIM15, TRIM26, HLA-L, HCG18, TRIM39-RPP21, TRIM39, RPP21
6p22.2	0.001569	LRRC16A, SCGN, HIST1H2AA, HIST1H2APS1, HIST1H2BA, SLC17A4, SLC17A1, SLC17A3, SLC17A2, TRIM38, HIST1H1A, HIST1H3A, HIST1H4A, HIST1H2AB, HIST1H3B, HIST1H4B, HIST1H2BB, HIST1H3C, HIST1H1C, HFE, HIST1H4C, HIST1H1T, HIST1H2AC, HIST1H2BC, HIST1H1E, HIST1H2BD, HIST1H2BE, HIST1H3D, HIST1H4D, HIST1H2AD, HIST1H2BF, HIST1H4E, HIST1H2BG, HIST1H2AE, HIST1H3E, HIST1H1D, HIST1H4F, HIST1H2BH, HIST1H3F, HIST1H4G, HIST1H3G, HIST1H2BI, HIST1H4H, BTN3A2, BTN2A2, BTN3A1, BTN2A3P, BTN3A3, BTN1A1, BTN2A1, HCG11, HMGN4, LOC285819, ABT1, ZNF322, GUSBP2, LINC00240, LOC100270746
6p22.3	0.001569	JARID2, DTNBP1, MYLIP, MIR4639, GMPR, ATXN1, FLJ23152, RBM24, CAP2, FAM8A1, NUP153, KIF13A, NHLRC1, TPMT, KDM1B, DEK, RNF144B, MIR548A1, hsa-mir-548a-1, ID4, MBOAT1, E2F3, CDKAL1, SOX4, LINC00340, LOC729177, PRL, HDGFL1, NRSN1, DCDC2, KAAG1, MRS2, GPLD1, ALDH5A1, KIAA0319, TDP2, ACOT13, C6orf62, GMNN, FAM65B, CMAHP
6p23	0.001569	SIRT5, NOL7, RANBP9, CCDC90A, RNF182, CD83
6p24.1	0.001569	ADTRP, HIVEP1, EDN1, PHACTR1, LOC100130357, TBC1D7, GFOD1
6p24.2	0.001569	C6orf52, PAK1IP1, TMEM14C, TMEM14B, MAK, GCM2, SYCP2L, ELOVL2, LOC100506409, C6orf228, ERVFRD-1, NEDD9, TMEM170B
6p24.3	0.001569	RREB1, SSR1, CAGE1, RIOK1, DSP, SNRNP48, BMP6, MUTED-TXNDC5, TXNDC5, PIP5K1P1, EEF1E1-MUTED, MUTED, EEF1E1, SCARNA27, SLC35B3, LOC100506207, HULC, TFAP2A, LOC100130275, LINC00518, GCNT2
6p25.1	0.001569	CDYL, RPP40, PPP1R3G, LYRM4, MIR3691, FARS2, NRN1, F13A1, LY86-AS1, LY86
6p25.2	0.001569	C6orf195, MYLK4, WRNIP1, SERPINB1, MGC39372, MIR4645, SERPINB9, SERPINB6, DKFZP686I15217, NQO2, RIPK1, BPHL, TUBB2A, LOC100507194, TUBB2B, PSMG4, SLC22A23, PXDC1, FAM50B, PRPF4B, FAM217A, C6orf201, ECI2
6p25.3	0.001569	DUSP22, IRF4, EXOC2, HUS1B, LOC285768, FOXQ1, FOXF2, FOXC1, GMDS, LOC100508120
14q21.1	0.001569	FOXA1, SSTR1, CLEC14A, LOC283547, SEC23A, GEMIN2, TRAPPC6B, PNN, MIA2, CTAGE5, LOC100288846, FBXO33, LRFN5
14q22.1	0.001569	ATL1, SAV1, NIN, ABHD12B, PYGL, TRIM9, TMX1, LOC283553, FRMD6, FRMD6-AS1, GNG2, C14orf166, NID2, PTGDR, PTGER2, TXNDC16, GPR137C, ERO1L, PSMC6, STYX, GNPNAT1, FERMT2, DDHD1
14q23.2	0.001569	HIF1A, HIF1A-AS2, SNAPC1, SYT16, FLJ43390, KCNH5, RHOJ, GPHB5, PPP2R5E, WDR89, SGPP1, SYNE2, hsa-mir-548h-1, ESR2
6p21.2	0.001626	CDKN1A, CPNE5, PPIL1, C6orf89, PI16, MTCH1, FGD2, PIM1, TMEM217, TBC1D22B, RNF8, FTSJD2, CCDC167, MIR4462, MDGA1, ZFAND3, BTBD9, GLO1, DNAH8, LOC100131047, GLP1R, SAYSD1, KCNK5, KCNK17, KCNK16, KIF6, DAAM2, MOCS1, FLJ41649, TDRG1, LRFN2

(cont.) Supplementary Table 3

List of significantly top 50 amplified genomic regions (cont.)

Cytoband	Adjusted p-value	Genes
20p12.3	0.00171	CDS2, PCNA-AS1, PROKR2, LOC100507629, LOC643406, LOC149837, GPCPD1, C20orf196, CHGB, TRMT6, MCM8, CRLS1, LRRN4, FERMT1, BMP2, HAO1, TMX4, PLCB1, PLCB4
20p13	0.00171	DEFB125, DEFB126, DEFB127, DEFB128, DEFB129, DEFB132, C20orf96, ZCCHC3, SOX12, NRSN2, TRIB3, RBCK1, TBC1D20, CSNK2A1, TCF15, SRXN1, SCRT2, C20orf54, FAM110A, ANGPT4, RSPO4, PSMF1, TMEM74B, C20orf202, RAD21L1, SNPH, FKBP1A-SDCBP2, SDCBP2, LOC100507495, FKBP1A, NSFL1C, SIRPB2, SIRPD, SIRPB1, SIRPG, LOC100289473, SIRPA, PDYN, STK35, TGM3, TGM6, SNORD119, SNRPB, ZNF343, TMC2, MIR1292, NOP56, SNORD110, hsa-mir-1292, IDH3B, SNORA51, SNORD56, SNORD57, SNORD86, EBF4, CPXM1, C20orf141, TMEM239, FAM113A, VPS16, PTPRA, GNRH2, MRPS26, OXT, AVP, LOC100134015, UBOX5, FASTKD5, ProSAPiP1, DDRGK1, ITPA, SLC4A11, C20orf194, ATRN, GFRA4, ADAM33, SIGLEC1, HSPA12B, C20orf27, CENPB, SPEF1, CDC25B, C20orf29, MAVS, PANK2, MIR103A2, MIR103B2, hsa-mir-103-2-as, hsa-mir-103-2, RNF24, SMOX, LOC728228, ADRA1D, PRNP, PRND, PRNT, RASSF2, SLC23A2, C20orf30, PCNA
6p12.1	0.001957	FBXO9, GCM1, ELOVL5, RPS16P5, GCLC, KLHL31, LRRC1, MLIP, TINAG, FAM83B, HCRTR2, GFRAL, HMGCLL1, BMP5, COL21A1, DST, BEND6, KIAA1586, ZNF451
14q23.3	0.002157	TEX21P, MTHFD1, AKAP5, ZBTB25, ZBTB1, HSPA2, PPP1R36, PLEKHG3, SPTB, CHURC1-FNTB, CHURC1, GPX2, RAB15, FNTB, MAX, MIR4706, LOC100506321, MIR4708, FUT8, LOC645431, hsa-mir-625, LINC00238, GPHN, FAM71D, MPP5, ATP6V1D, EIF2S1, PLEK2
17q22	0.002511	KIF2B, LOC100506650, TOM1L1, COX11, STXBP4, HLF, MMD, TMEM100, PCTP, ANKFN1, NOG, C17orf67, DGKE, MTVR2, MIR3614, TRIM25, COIL, SCPEP1, RNF126P1, AKAP1, MSI2, MRPS23, CUEDC1, VEZF1, SRSF1, DYNLL2, MSX2P1, OR4D1, OR4D2, EPX, MKS1, LPO, MPO, BZRAP1, LOC100506779, MIR142, MIR4736, hsa-mir-142, SUPT4H1, RNF43, HSF5, MTMR4, SEPT4, C17orf47, TEX14, RAD51C, PPM1E, TRIM37, SKA2, MIR454, hsa-mir-454, MIR301A, PRR11, hsa-mir-301a, SMG8, GDPD1, YPEL2, MIR4729
17q23.1	0.002511	DHX40, CLTC, PTRH2, VMP1, MIR21, hsa-mir-21, TUBD1, RPS6KB1, RNFT1, TBC1D3P1-DHX40P1, HEATR6, MIR4737, LOC645638, LOC653653, CA4, USP32
6p12.2	0.002511	MIR133B, MIR206, hsa-mir-133b, hsa-mir-206, IL17A, IL17F, MCM3, PAQR8, EFHC1, TRAM2, LOC730101, TMEM14A, GSTA7P, GSTA2, GSTA1, GSTA5, GSTA3, GSTA4, ICK
6p12.3	0.002511	CYP39A1, SLC25A27, TDRD6, PLA2G7, LOC100287718, MEP1A, GPR116, GPR110, TNFRSF21, CD2AP, GPR111, GPR115, OPN5, PTCHD4, MUT, CENPQ, GLYATL3, C6orf141, RHAG, CRISP2, CRISP3, PGK2, CRISP1, DEFB133, DEFB113, DEFB114, DEFB110, DEFB112, TFAP2D, TFAP2B, PKHD1
6p21.1	0.002511	UNC5CL, TSPO2, APOBEC2, C6orf130, NFYA, LOC221442, TREML1, TREM2, TREML2, TREML3, TREML4, TREML2P1, TREM1, NCR2, FOXP4, MIR4641, MDFI, TFEB, PGC, FRS3, PRICKLE4, TOMM6, USP49, MED20, BYSL, CCND3, TAF8, C6orf132, GUCA1A, GUCA1B, MRPS10, TRERF1, UBR2, PRPH2, ATP6V0CP3, TBCC, KIAA0240, RPL7L1, C6orf226, CNPY3, PTCRA, GNMT, PEX6, PPP2R5D, KLHDC3, MEA1, CUL7, KLC4, MRPL2, RRP36, PTK7, SRF, CUL9, C6orf108, TTBK1, SLC22A7, CRIP3, ZNF318, ABCC10, DLK2, TJAP1, LRRC73, POLR1C, YIPF3, XPO5, POLH, GTPBP2, MAD2L1BP, RSPH9, MRPS18A, VEGFA, LOC100132354, C6orf223, MRPL14, TMEM63B, CAPN11, SLC29A1, HSP90AB1, MIR4647, NFKBIE, SLC35B2, TMEM151B, TCTE1, AARS2, SPATS1, CDC5L, MIR4642, SUPT3H, MIR586, hsa-mir-586, RUNX2, CLIC5, ENPP4, ENPP5, RCAN2
14q21.2	0.002511	FSCB, C14orf28, KLHL28, FAM179B, PRPF39, FKBP3, SNORD127, FANCM, MIS18BP1, RPL10L
6p21.32	0.002695	LOC100507547, PPT2-EGFL8, PPT2, PRRT1, AGER, AGPAT1, EGFL8, PBX2, RNF5P1|chr6, RNF5, GPSM3, NOTCH4, C6orf10, HCG23, BTNL2, HLA-DRA, HLA-DRB5, HLA-DRB6, HLA-DRB1, HLA-DQA1, HLA-DQB1, HLA-DQA2, HLA-DQB2, HLA-DOB, TAP2, LOC100507463, PSMB8, TAP1, PSMB9, LOC100294145, HLA-DMB, HLA-DMA, BRD2, HLA-DOA, HLA-DPA1, HLA-DPB1, HLA-DPB2, COL11A2, RXRB, HSD17B8, MIR219-1, RING1, SLC39A7, hsa-mir-219-1, HCG25, VPS52, RPS18, B3GALT4, WDR46, PFDN6, RGL2, TAPBP, DAXX, ZBTB22, KIFC1, CUTA, PHF1, SYNGAP1, ZBTB9
11q13.2	0.003062	KLC2, RAB1B, CNIH2, TMEM151A, YIF1A, CD248, RIN1, BRMS1, B3GNT1, SLC29A2, NPAS4, MRPL11, PELI3, DPP3, BBS1, ZDHHC24, ACTN3, CTSF, CCDC87, CCS, RBM14-RBM4, RBM14, RBM4, RBM4B, SPTBN2, C11orf80, PC, RCE1, LRFN4, hsa-mir-3163, C11orf86, SYT12, RHOD, KDM2A, ADRBK1, ANKRD13D, SSH3, LOC100130987, POLD4, CLCF1, PPP1CA, RAD9A, TBC1D10C, CARNS1, RPS6KB2, CORO1B, PTPRCAP, GPR152, CABP4, TMEM134, AIP, CDK2AP2, PITPNM1, CABP2, GSTP1, NDUFV1, DOC2GP, NUDT8, TBX10, ACY3, ALDH3B2, FAM86C2P, UNC93B1, ALDH3B1, NDUFS8, MIR4691, TCIRG1, CHKA, SUV420H1, C11orf24, LRP5, PPP6R3
14q24.2	0.003062	LOC100289511, SRSF5, SLC10A1, SMOC1, SLC8A3, ADAM21P1, COX16, SYNJ2BP-COX16, SYNJ2BP, ADAM21, C14orf55, ADAM20, MED6, TTC9, MAP3K9, PCNX, SNORD56B, LOC145474, SIPA1L1, RGS6, DPF3, DCAF4, ZFYVE1, RBM25, PSEN1, PAPLN, NUMB
6p21.33	0.003488	HLA-E, GNL1, PRR3, ABCF1, MIR877, hsa-mir-877, PPP1R10, MRPS18B, ATAT1, C6orf136, DHX16, PPP1R18, NRM, MDC1, TUBB, FLOT1, IER3, DDR1, MIR4640, GTF2H4, VARS2, SFTA2, DPCR1, MUC21, MUC22, HCG22, C6orf15, CDSN, PSORS1C1, CCHCR1, PSORS1C2, TCF19, POU5F1, PSORS1C3, HCG27, HLA-C, HLA-B, MICA, HCP5, HCG26, MICB, MCCD1, ATP6V1G2-DDX39B, ATP6V1G2, DDX39B, LST1, LTA, LTB, NCR3, NFKBIL1, SNORD117, SNORD84, TNF, ABHD16A, AIF1, APOM, BAG6, C6orf25, C6orf47, CLIC1, CSNK2B, DDAH2, GPANK1, LSM2, LY6G5B, LY6G5C, LY6G6C, LY6G6D, LY6G6E, LY6G6F, MIR4646, MSH5-SAPCD1, MSH5, PRRC2A, SAPCD1, SNORA38, VARS, VWA7, HSPA1L, HSPA1A, C6orf48, HSPA1B, SNORD48, SNORD52, NEU1, SLC44A4, EHMT2, ZBTB12, C2, CFB, RDBP, MIR1236, SKIV2L, hsa-mir-1236, DOM3Z, STK19, C4A, C4B, LOC100293534, CYP21A1P, CYP21A2, TNXA, TNXB, ATF6B, FKBPL
18p11.22	0.006123	RAB12, CCDC165, NDUFV2, ANKRD12, TWSG1, RALBP1, PPP4R1, RAB31, TXNDC2, VAPA, APCDD1, NAPG, PIEZO2
14q24.1	0.007394	TMEM229B, PLEKHH1, PIGH, ARG2, VTI1B, RDH11, RDH12, ZFYVE26, RAD51B, ZFP36L1, ACTN1, DCAF5, EXD2, GALNTL1, ERH, SLC39A9, PLEKHD1, C14orf162, KIAA0247
5p15.33	0.007574	PLEKHG4B, LRRC14B, CCDC127, SDHA, PDCD6, AHRR, C5orf55, EXOC3, PP7080, SLC9A3, MIR4456, CEP72, TPPP, ZDHHC11, BRD9, TRIP13, LOC100506688, NKD2, SLC12A7, MIR4635, SLC6A19, SLC6A18, TERT, CLPTM1L, MIR4457, SLC6A3, LPCAT1, SDHAP3, LOC728613, MIR4277, hsa-mir-4277, MRPL36, NDUFS6, IRX4, C5orf38, IRX2, LOC285577, IRX1
17q25.1	0.00879	SSTR2, COG1, FAM104A, C17orf80, CPSF4L, CDC42EP4, SDK2, LINC00469, LOC400620, RPL38, MGC16275, TTYH2, DNAI2, KIF19, BTBD17, GPR142, GPRC5C, CD300A, CD300LB, CD300C, C17orf77, CD300LD, CD300E, RAB37, CD300LF, MIR3615, SLC9A3R1, NAT9, TMEM104, GRIN2C, FDXR, FADS6, C17orf28, OTOP2, OTOP3, USH1G, CDR2L, ICT1, ATP5H, KCTD2, SLC16A5, ARMC7, NT5C, HN1, SUMO2, GGA3, NUP85, LOC100287042, MIF4GD, MRPS7, SLC25A19, GRB2, MIR3678, KIAA0195, CASKIN2, TSEN54, LLGL2, MYO15B, RECQL5, C17orf109, C17orf110, SAP30BP, ITGB4, GALK1, H3F3B, MIR4738, UNK, UNC13D, WBP2, TRIM47, TRIM65, MRPL38, FBF1, ACOX1, TEN1-CDK3, TEN1, CDK3, EVPL, SRP68, GALR2, EXOC7, ZACN, FOXJ1, LOC100507218, RNF157, FAM100B, QRICH2, PRPSAP1, SPHK1, UBE2O, AANAT, RHBDF2, CYGB, PRCD, LOC100507246, SNORD1C, SNORD1A, SNORD1B, ST6GALNAC2, ST6GALNAC1, MXRA7, JMJD6, METTL23, SRSF2, MFSD11, MIR636, hsa-mir-636
17q25.2	0.00879	MGAT5B, LINC00338, SCARNA16, SEC14L1, SEPT9
17q25.3	0.00879	MIR4316, hsa-mir-4316, LOC100507351, FLJ45079, TNRC6C, LOC100131096, TMC6, TMC8, C17orf99, SYNGR2, TK1, AFMID, BIRC5, TMEM235, SOCS3, PGS1, DNAH17, CYTH1, USP36, TIMP2, LOC100653515, LGALS3BP, CANT1, C1QTNF1, LOC100507410, ENGASE, RBFOX3, MIR4739, ENPP7, CBX2, CBX8, CBX4, TBC1D16, CCDC40, GAA, EIF4A3, CARD14, SGSH, SLC26A11, RNF213, LOC100294362, ENDOV, MIR4730, NPTX1, RPTOR, CHMP6, FLJ90757, BAIAP2, AATK, MIR3065, MIR338, MIR657, hsa-mir-3065, hsa-mir-338, hsa-mir-657, MIR1250, hsa-mir-1250, AATK-AS1, AZI1, C17orf56, C17orf89, SLC38A10, LINC00482, TMEM105, BAHCC1, MIR4740, MIR3186, hsa-mir-3186, ACTG1, FSCN2, C17orf70, NPLOC4, TSPAN10, PDE6G, C17orf90, CCDC137, ARL16, HGS, MRPL12, SLC25A10, GCGR, FAM195B, PPP1R27, P4HB, ARHGDIA, ALYREF, ANAPC11, NPB, PCYT2, MAFG, SIRT7, MAFG-AS1, PYCR1, MYADML2, NOTUM, ASPSCR1, STRA13, LRRC45, DCXR, RAC3, RFNG, GPS1, DUS1L, FASN, CCDC57, SLC16A3, CSNK1D, CD7, SECTM1, TEX19, UTS2R, C17orf101, HEXDC, C17orf62, NARF, FOXK2, WDR45L, RAB40B, FN3KRP, FN3K, TBCD, ZNF750, B3GNTL1, METRNL, FLJ43681
14q31.3	0.00879	FLRT2, LOC283585, GALC, GPR65, LOC283587, KCNK10, SPATA7, PTPN21, ZC3H14, EML5, TTC8, FOXN3
14q32.12	0.00879	SMEK1, CATSPERB, TC2N, FBLN5, TRIP11, ATXN3, CPSF2, NDUFB1, SLC24A4, RIN3, LGMN, GOLGA5, CHGA, ITPK1, ITPK1-AS1, C14orf109, MOAP1, C14orf142, UBR7, BTBD7, UNC79, COX8C, PRIMA1, FAM181A-AS1, FAM181A, ASB2, LINC00521, OTUB2, DDX24, IFI27L1, IFI27, IFI27L2, PPP4R4

Supplementary Table 4: Pathway enrichment analysis for differentially expressed genes in tumors with low or high immune-ITH

Selective list of pathways enriched in tumours with low immune-ITH

Pathway	Gene name	Benjamini
Mitochondrion	*ETNPPL, MRPS36, UQCRC1, CPT2, AMT, ECHDC2, CLYBL, NDUFAF1, PDHB, AUH, MCCC2, SLC25A20, NDUFS5, ALAS1, MSRA, MCEE, MRPL32, HADH, DHTKD1, HMGCL, DNAJC19, OMA1, MRPL35, AADAT, ALDH6A1, ACAA2, SUOX, SLC25A4, CYP11A1, ALDH5A1, NUDT6, OTC, COQ9, CBR4, DECR1, COX6C, COQ4, ACADVL, ALDH7A1, ABAT, ATPAF1, MRPL46, PCCA, PRODH, HSD17B10, NDUFB8, CA5A, COX7B, ECHS1, AASS, RSAD1, SFXN2, ATP5G1, PIN4, ACAT1, TK2, RPP14, GLS2, ACSL1, IVD, DHODH, ETFDH, DMGDH, BDH1, FH, FBXO7, GIMAP5, C14ORF159, MRPS25, MAOB, C21ORF33, ACACB, CPS1, VDAC3, SIRT3, DBT, GLYAT, SARM1, PDE2A, HMGCS2, SDHD, ALDH2, COQ10A, HIBCH, ACAD11, SCP2, PHYKPL, TRIT1*	1.05E-14
Glycoprotein	*ADCY4, FAM20A, CXORF36, IGFBP7, CRHBP, TACR1, AQP1, MMRN2, ST6GALNAC6, EDNRB, ST6GALNAC3, ITPRIP, ROBO4, ESAM, TIE1, BOC, RAMP3, RAMP2, CTSZ, BTNL9, EVA1C, CRTAC1,* ***COL25A1, CLEC1A,*** *MTMR14, THBD, LAMC3, H6PD, CD300LG,* ***ADAMTS1,*** *JAM2, EMP1,* ***ADAMTS4,*** *GPR182, EMCN, HLA-DRB1, LRIG1, PLVAP, GPR4, CDH5, SLCO2A1, RGMA,* ***CLEC2B,*** *TEK, GALNT15, LRRC70, HBB, PTPRB, IL1RL1, FLT4, PCDH12, TSPAN18, KDR, ITGA9, CCL14, APOL1, CLEC3B, NOTCH4, ENG, SCN4A, CLEC14A, IGFBP4*	5.79E-06
Fatty acid metabolism	***ACAA2, CPT2,*** *PRKAG2,* ***ECHDC2,*** *ECHS1,* ***CBR4,*** *ACACB, DECR1, RPP14, ACOX3, ACADVL, ACSL1, HADH, SLC27A2,* ***ACAA1***	4.13E-05
Peroxisome	*EPHX2, PEX11G, PIPOX, ACOX3, ACSL1, LONP2, NUDT7, PXMP2, DAO, CAT, ACAD11, SLC27A2, SCP2, HMGCL, ACAA1*	4.92E-05
Pyridoxal phosphate	*ETNPPL, AADAT, ALAS1, CTH, GOT1, SDS, SEPSECS, ABAT, GPT2, TAT, PHYKPL, CBS*	9.45E-05
Flavoprotein	*STEAP4, MAOB, PIPOX, ACOX3, ACADVL, FMO4, MMACHC, IVD, DHODH, ETFDH, DMGDH, DAO, ACAD11, ACAD10, PRODH*	5.58E-04
Microsome	*CYP2U1, CYP2B6, CYP2C8, EPHX1, CYP26A1, CYB5A, FMO4, CYP4A11, ACSL1, CYP4A22, CYP2A6, UGT2B10, CYP8B1, UGT2B15, MGST2*	7.72E-04

Selective list of pathways enriched in tumours with high immune-ITH

Pathway	Gene name	Benjamini
Cell cycle	***CDK19,*** *KIF23,* ***KIFC1,*** *E2F3, PARD3, HAUS6, TICRR, LIN9, DBF4, ANLN, AURKA, CEP55, CD2AP, KIF13A, SPC25,* ***MCM8, MCM7, CSNK2A1,*** *MDC1, CENPA, HJURP, INCENP, BUB1, RALA, MASTL, ERCC6L, KIF2A, PRPF40A, KHDRBS1, ANAPC1, DLGAP5, UBE2I, RACGAP1, PPP1CC, HMGA2, ECT2, SUV39H2, RNF8, NEDD1, FAM64A, MAD2L1, CENPW, ANAPC7, MAPRE1, CUL4B, NUP43, DSCC1*	2.41E-07
Nucleotide-binding	*ABCF1, KIF23, HSP90AB1, CDK19, KIFC1, PRPF4B, TUBB2B, STK38, NARS, FIGNL1, TUBB2A, TTLL4, TTK, AURKA, MTHFD1L, ATP2B1, PKM, KIF13A, DDX27, HSPH1, ACTR2, MCM8, MCM7, CSNK2A1, MAP3K9, ORC4, RAB23, RALA, MKKS, MASTL, KIF2A, GTPBP2, TYRO3, ARL1, GTPBP4, UBE2A, DARS, CSNK1G1, MYO3B, OLA1, UBE2I, CCT6A, SRPK1, UBE2N, UBE2O, ATP9A, SMARCAL1, FARSB, ACVR1, GATC, ARL4A, ALDH18A1, BLM, DNAH14, HK2, RTKN, EPHA10, RIOK1, VARS, TUBB, DGKB, CHD1L, BUB1, DYRK2, TRIP13, ERCC6L, PDK1, MOCS3, TCP1, ATP1A1, GUCY2C, CCT7, HSP90B1, CCT5, CCT4, MLK4, UBA2, NLRP11, APAF1, HSPD1, AACS*	2.63E-04
Centromere	*CENPL, PPP1CC, CENPI, SUV39H2, SPC25, MAD2L1, HJURP, CENPA, INCENP, BUB1, CENPW, NUP107, NUP43, ERCC6L, PHF6*	6.50E-04
Chaperone	***PTGES3,*** *HSP90AB1,* ***TCP1,*** *VBP1, PDIA6,* ***CCT6A, CCT7, CCT5,*** *HSP90B1,* ***CCT4,*** *HJURP, TBCC, MKKS, HSPD1, CHORDC1, TOMM34*	0.00673
Microtubule	*GAS2L3, KIF23, KIFC1, HAUS6, TUBB2B, TUBB2A, DNAH14, TTLL4, AURKA, KIF13A, TUBB, KATNA1, INCENP, TUBA3E, APPBP2, KIF2A, CAPN6, KIF5A, KIF16B, RACGAP1, EML6, DCTN1, TRIM54, TUBD1, MAPRE1, SPAST, DYNC1I2*	0.00976
Transcription	*ZNF85, ISX, E2F3, ZKSCAN8, TAF1A, E2F5, ZNF675, TBP, MED23, ZIC1, ZKSCAN3, MED20, SOHLH2, ZBTB38, ESF1, NONO, KDM1B, ZNF184, EPC2, CSNK2A1, ZNF738, MAP3K9, ZNF681, PRIM2, YAP1, KHDRBS1, ZNF100, ZNF280C, SNAPC1, ZNF92, ZNF142, ZNF189, GTF2H3, HMGA2, HMGA1, TOX3, CD3EAP, EYA1, HIF1A, NCOA3, TRIM33, ZNF714, GTF2IRD1, ZNF239, KHSRP, CNOT11, ZNF431, ZNF480, ZNF713, LRPPRC, XRN2, ZNF844, ZNF273, ZFP64, ZBTB9, WBSCR22, NFYA, MYBL2, POLR2D, ATF1, CDYL, ZNF708, ZNF697, ZNF74, ACTL6A, TCF3, PLAG1, POLR3F, ZNF28, JARID2, TRIM28, TBX4, ZNF66, TRIM27, SMAD3, TEAD3, TRIM24, POLR3C, SUV39H2, ZNF664, ATXN1, HDAC2, ZSCAN31, ILF2, BCORL1, DR1, SP3, CPNE1, ZNF318, NELFE, PBX2, PHF6*	0.00971

Supplementary Table 5: Univariate analysis of clinical and biological variables

	Univariate analysis^Ψ	
**Variable**	**HR (CI 95%)**	**p-value**
DNA-ITH #	2.06 (0.68-6.22)	0.20
RNA-ITH #	1.44 (0.48-4.33)	0.52
Immune-ITH #	4.69 (1.28-17.15)	***0.02***
Total neoantigen #	1.00 (0.99-1.02)	0.71
Subclonal neoantigen #	1.01 (1-1.03)	0.08
% $TNF\alpha^{+}IFN\gamma^{+}CD3^{+}$ T cells #	1.19 (0.40-3.53)	0.76
$CD3^{+}$ T cell density (by IHC) #	1.50 (0.50-4.50)	0.47
Fibrosis stage (F0-F4) *	0.96 (0.62-1.47)	0.84
Stage, I+II/III $	39.96 (4.69-340.71)	***0.00075***
Grade, I/II/III/IV ^	1.46 (0.73-2.89)	0.28
Tumour size, cm (< or ≥ 5cm)	8.26 (1.82-37.51)	***0.0062***
Microvascular invasion, Y/N	7.04 (1.88-26.45)	***0.0038***
HBV infection, Y/N	0.73 (0.25-2.12)	0.56
Gender, F/M	0.70 (0.20-2.53)	0.59
Race Δ	2.142 (1.29-3.57)	***0.0034***
Age (years), 47-82	1.03 (0.96-1.10)	0.46
Tumour multiplicity, Y/N	1.45 (0.51-4.06)	0.48
AFP level (ng/ml), 1.5- >60,500	1 (1-1)	0.13

Footnote:

Ψ Cox proportional hazards regression analysis with hazard ratio (HR) and two-tailed P values adjusted for multiple testing

≤ versus > median

* Metavir Scoring system

$ TNM staging version 8

^ Edmondson

HBV: Hepatitis B

Y/N: Yes/No

F/M: female/male

AFP: Alpha-fetoprotein

Δ Chinese= 0, Malay= 1, Indian= 2, Indonesian= 3, others= 4

a

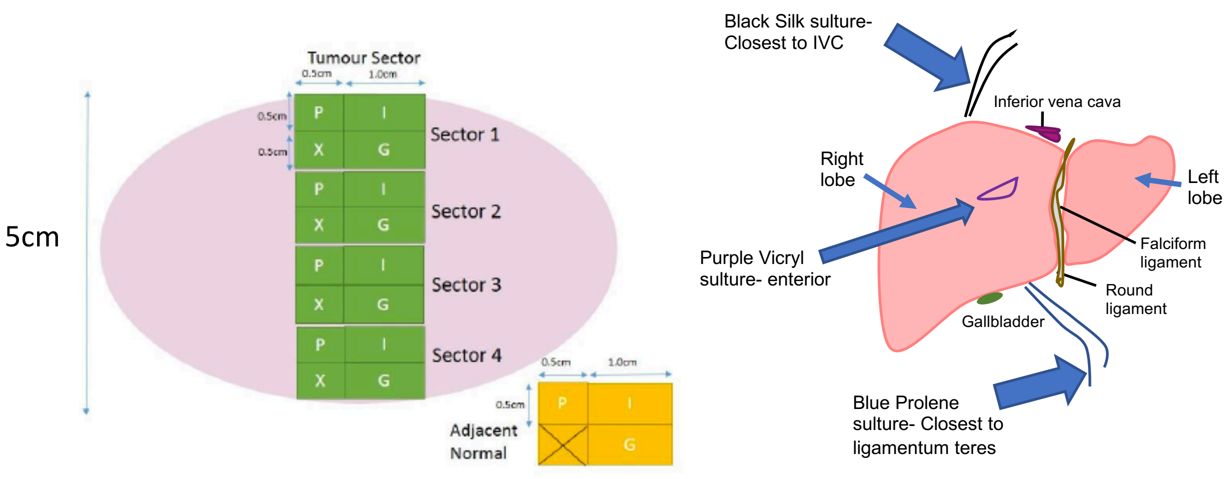

b

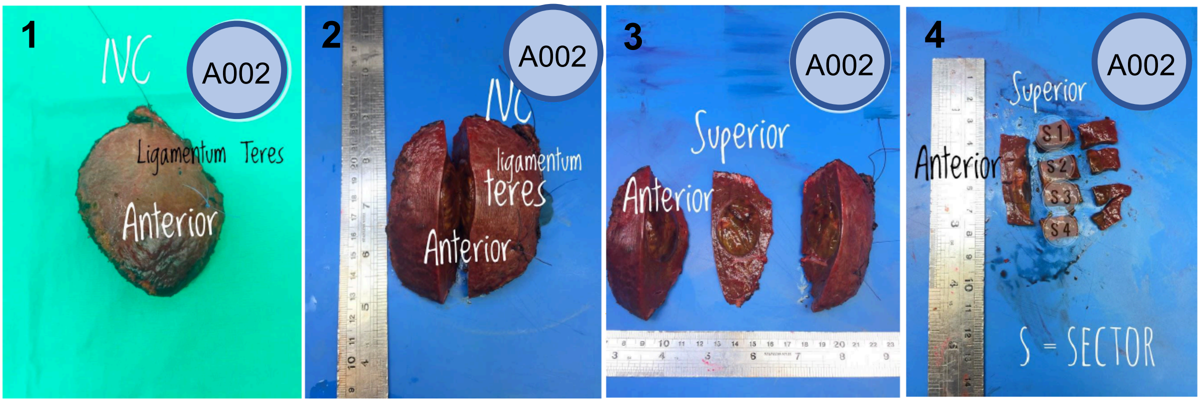

Supplementary Fig. 1. Tumour sectors collection protocol.

a Left, Illustration of multiple tumour sectors sampling each of which is of size 1 cm (width) x 0.5 cm (height) x 0.5 cm (thickness) for P=PDP (patient-derived progenitor cell), X=PDX (patient-derived xenograft), G=Genomics, I=Immunology. Each sector is clearly separated by a gap between them. Note: no PDX for adjacent normal tissue. Right, Illustration stitches for orientation of tumour: Black silk suture for the part of the tumour closest to the supra hepatic IVC; Blue prolene suture for part of the tumour closest to the ligamentum teres. Note: Black and blue sutures will be used to obtain the cranial-caudal alignment of the specimen relative to patient and Purple vicryl suture for anterior surface in vivo.

b A representative tumour from patient ID A002 following the sampling protocol as illustrated in (a).

a

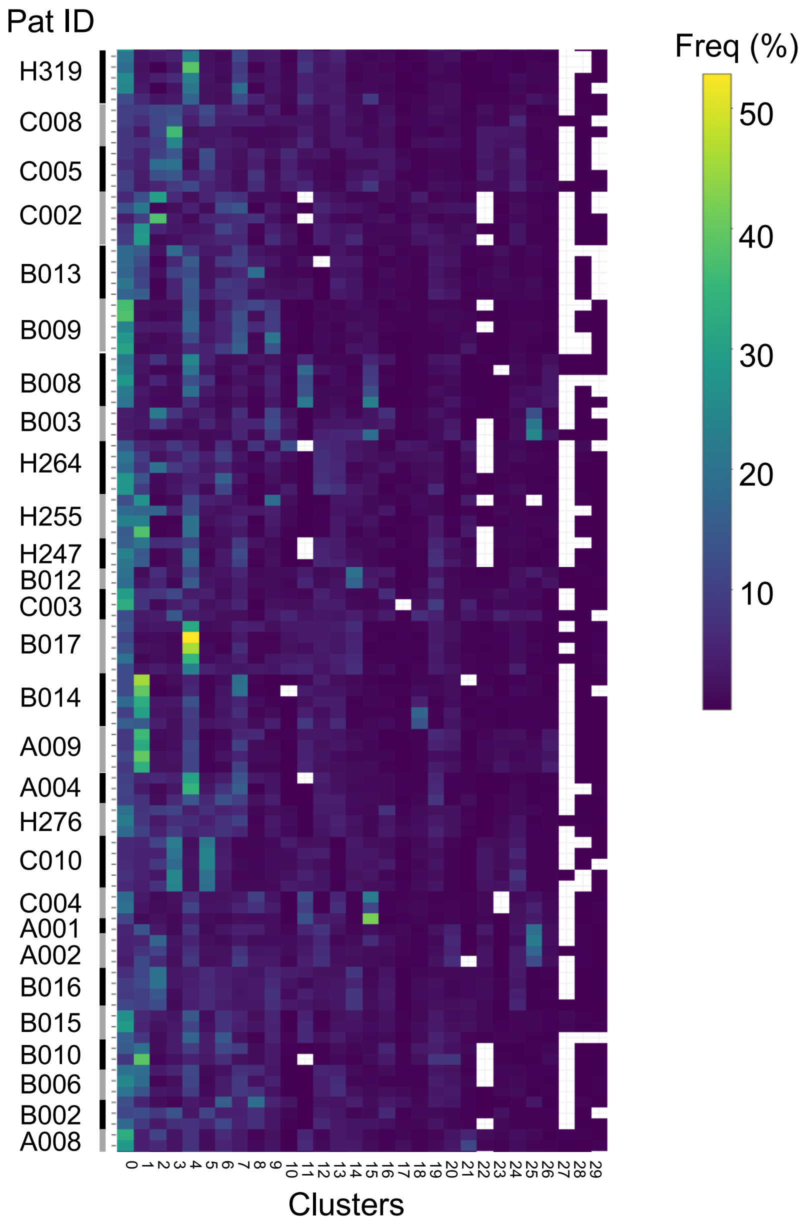

Supplementary Fig. 2: Immune clusters frequencies across different tumour sectors from each patient.

a Heatmap showing frequencies (Freq, %) of each cluster: 0-29 (x-axis) from Phenograph clustering in 2 to 5 sectors from each tumour with patient (pat) ID as listed on the y-axis.

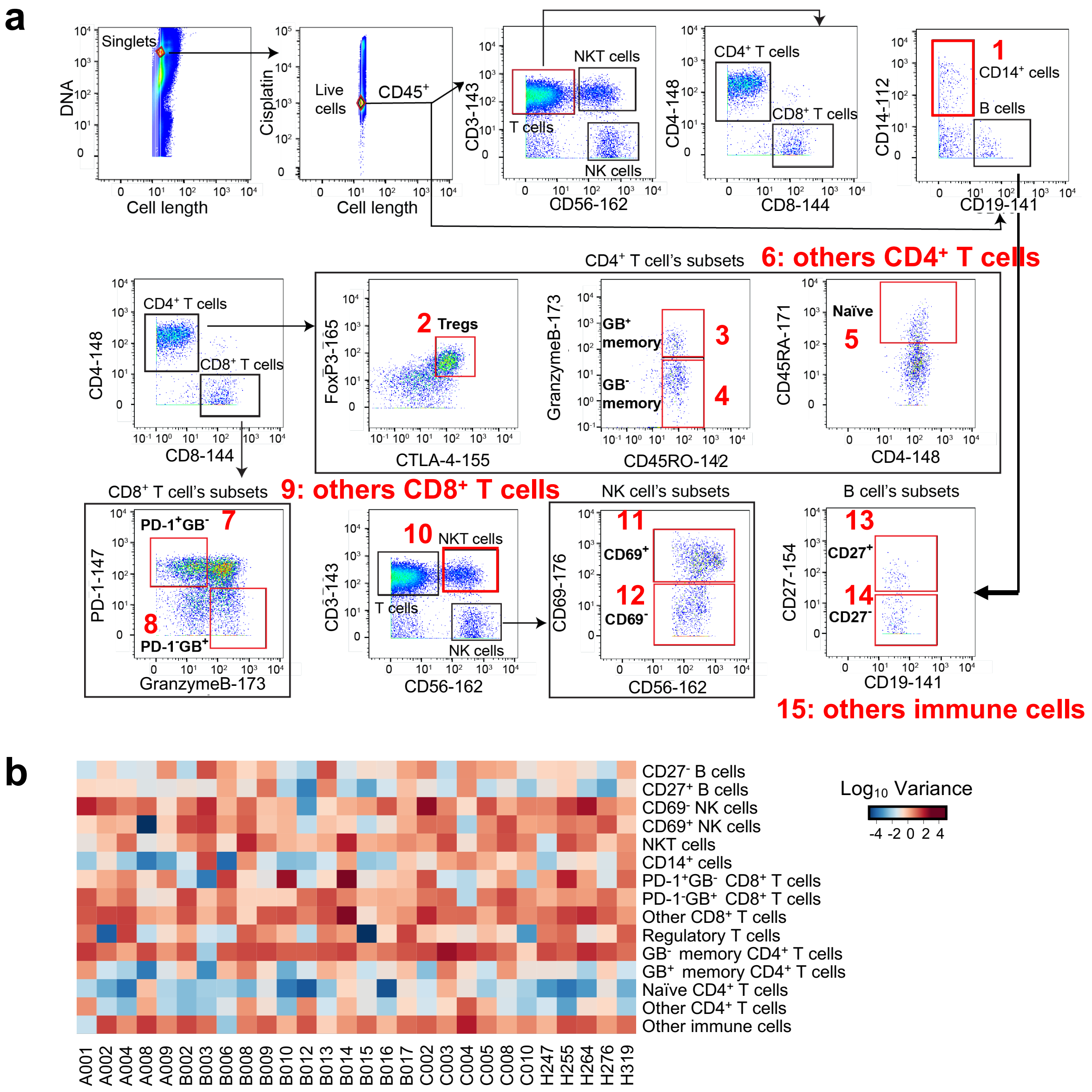

Supplementary Fig. 3: Immune subsets analysis from TILs acquired by CyTOF.

a Representative dot plots showing the manual gating of 15 immune subsets using FlowJo. Singlets were gated out as cells positive for iridium DNA intercalator (DNA$^+$, y-axis) of a specific cell length (x-axis). All live immune cells were gated for Cisplatin(Live/dead dye)$^-$ CD45$^+$ populations. 15 immune subsets (red boxes) were gated using their respective immune markers. **1:** CD14$^+$ macrophages; CD4$^+$ T cells subsets: **2:** Foxp3$^+$ CTLA-4$^+$ CD4$^+$ Treg, **3/4:** GB$^{+/-}$ CD45RO$^+$CD4$^+$ memory, **5:** CD45RA$^+$CD4$^+$ naïve T cells, **6:** other CD4+ T cells: Total CD4$^+$ T cells - (2,3,4 &5); CD8$^+$ T cells subsets: **7:** PD-1$^+$GB$^-$ exhausted CD8$^+$ T cells, **8:** PD-1$^-$GB$^+$ activated CD8$^+$ T cells, **9:** others CD8 T cells: Total CD8$^+$ T cells - (8 & 9); **10:** CD56$^+$CD3$^+$ NKT cells; **11/12:** CD69$^+$/CD69$^-$CD56$^+$ NK cells; **13/14:** CD27$^+$/CD27$^-$CD19$^+$ B cells; and **15:** others: total CD45$^+$ immune cells - (1-14).

b Heatmap showing Log$_{10}$ variance of the proportion of 15 immune subsets across different tumour sectors from all 28 HCC patients.

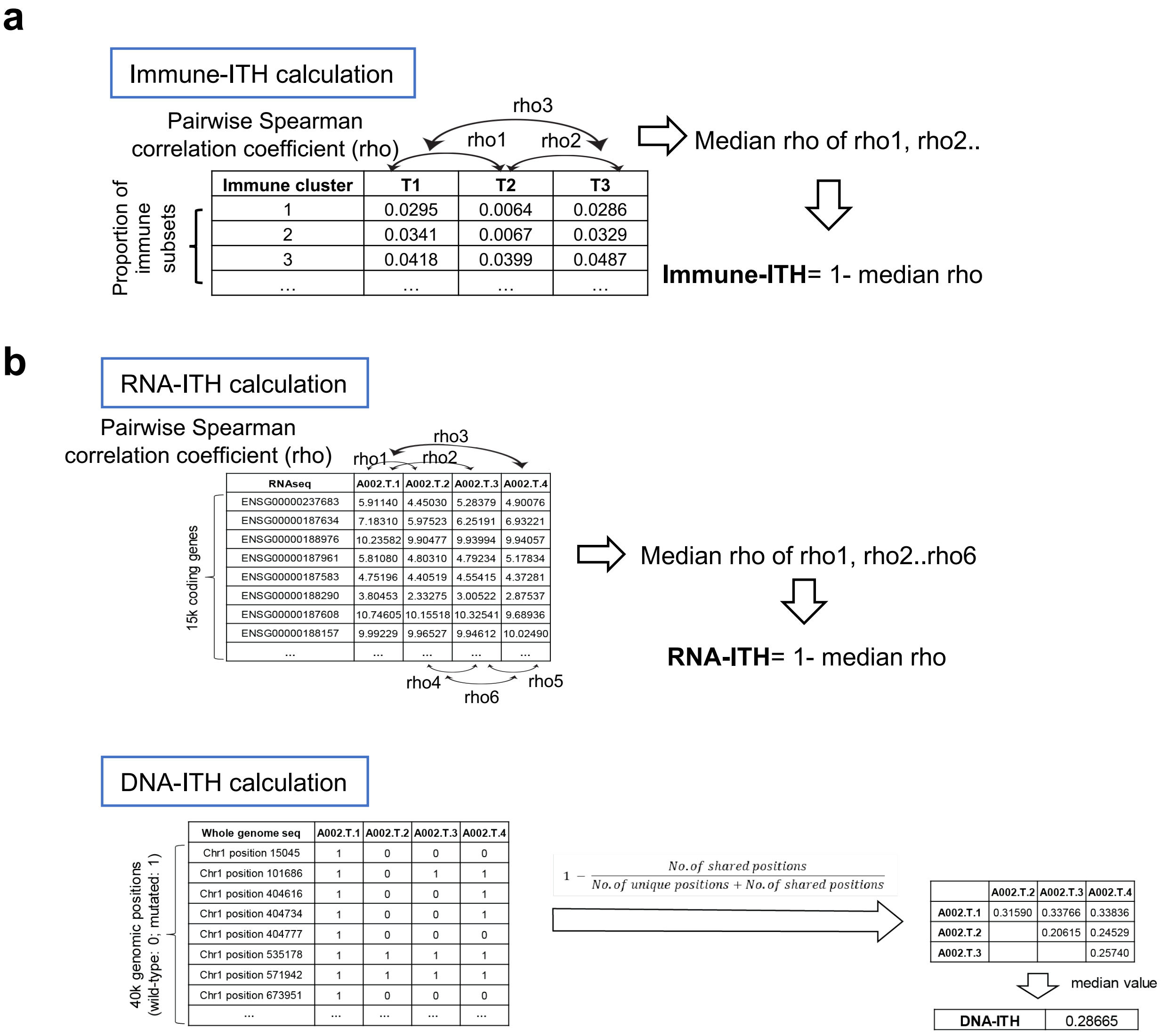

Immune cluster	T1	T2	T3
1	0.0295	0.0064	0.0286
2	0.0341	0.0067	0.0329
3	0.0418	0.0399	0.0487
...	...	...	...

RNAseq	A002.T.1	A002.T.2	A002.T.3	A002.T.4
ENSG00000237683	5.91140	4.45030	5.28379	4.90076
ENSG00000187634	7.18310	5.97523	6.25191	6.93221
ENSG00000188976	10.23582	9.90477	9.93994	9.94057
ENSG00000187961	5.81080	4.80310	4.79234	5.17834
ENSG00000187583	4.75196	4.40519	4.55415	4.37281
ENSG00000188290	3.80453	2.33275	3.00522	2.87537
ENSG00000187608	10.74605	10.15518	10.32541	9.68936
ENSG00000188157	9.99229	9.96527	9.94612	10.02490
...	...	...	...	...

Whole genome seq	A002.T.1	A002.T.2	A002.T.3	A002.T.4
Chr1 position 15045	1	0	0	0
Chr1 position 101686	1	0	1	1
Chr1 position 404616	1	0	0	1
Chr1 position 404734	1	0	0	1
Chr1 position 404777	1	0	0	0
Chr1 position 535178	1	1	1	1
Chr1 position 571942	1	1	1	1
Chr1 position 673951	1	0	0	0
...	...	...	...	...

	A002.T.2	A002.T.3	A002.T.4
A002.T.1	0.31590	0.33766	0.33836
A002.T.2		0.20615	0.24529
A002.T.3			0.25740

DNA-ITH	0.28665

Supplementary Fig. 4: Quantification and analysis of the degree of intratumoural heterogeneity (ITH).

a The immune--ITH were calculated as 1 minus Spearman correlation coefficient (rho) of the relative proportions of 15 immune subsets, which indicates the degree of heterogeneity for each individual tumour.

b RNA-ITH were calculated as 1 minus Spearman correlation coefficient (rho) of the relative RNA expression values of all genes from pairwise comparisons across all tumour sectors per patient. DNA-ITH was calculated as ratio of the number of unique DNA mutations to the total number of DNA mutations.

a and b Their median values were taken as the patient-level immune, transcriptomic and genomic-ITH scores, respectively.

a

No.	Patient ID	Distance metrics		Immune-ITH
		Spearman's correlation	Euclidean	
1	A008	0.00714	2.00000	Low
2	C010	0.02323	3.58869	Low
3	B010	0.02500	3.74166	Low
4	B015	0.03399	4.52770	Low
5	B016	0.03618	4.49579	Low
6	A002	0.03750	4.40957	Low
7	H276	0.04286	4.89898	Low
8	A009	0.05719	5.80944	Low
9	B003	0.07143	6.32456	Low
10	B012	0.08311	6.81909	Low
11	C004	0.08571	6.92820	Low
12	B002	0.09286	7.21110	Low
13	C003	0.09643	7.34847	Low
14	C005	0.09821	7.41344	Low
15	A004	0.11071	7.87401	High
16	H247	0.11071	7.87401	High
17	H319	0.11964	8.16679	High
18	B006	0.13929	8.83176	High
19	B017	0.15231	9.22473	High
20	B008	0.17143	9.79583	High
21	C002	0.17321	9.77839	High
22	H255	0.17500	9.54686	High
23	C008	0.18052	10.13437	High
24	B009	0.23091	11.36868	High
25	A001	0.25000	11.83216	High
26	B013	0.26810	12.24607	High
27	B014	0.29107	12.68356	High
28	H264	0.34643	13.92839	High
	**MEDIAN**	**0.10446**	**7.64372**	

Legend: Immune-ITH — Low (green); High (red)

Supplementary Fig. 5: Robust immune-intratumoural heterogeneity (ITH) score
a Immune-ITH scores and grouping using Spearman's correlation and Euclidean distance metrics. Low immune-ITH < Median and High immune-ITH > Median.

a

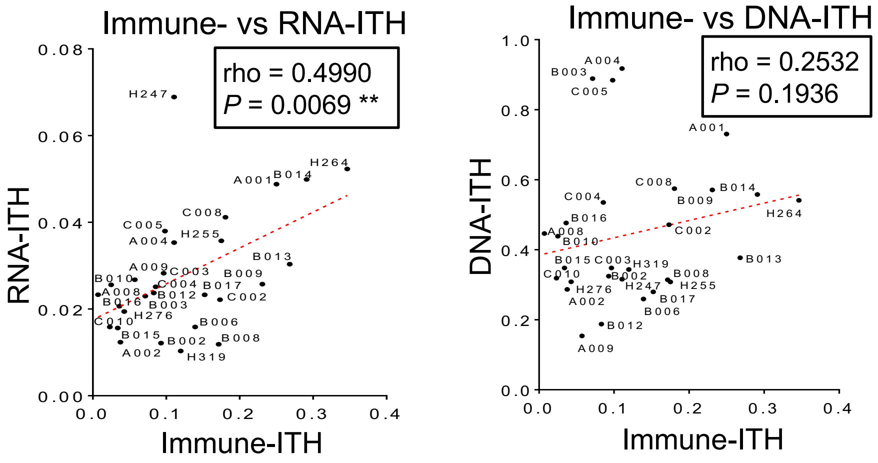

Supplementary Fig.6: Quantification and analysis of the degree of intratumoural heterogeneity (ITH) for each patient.

a, Correlation of immune-ITH with RNA (transcriptomic) or DNA (genomics)-ITH. Data analysis using Spearman's rho, * P<0.05 and **P <0.01. n=28 patients.

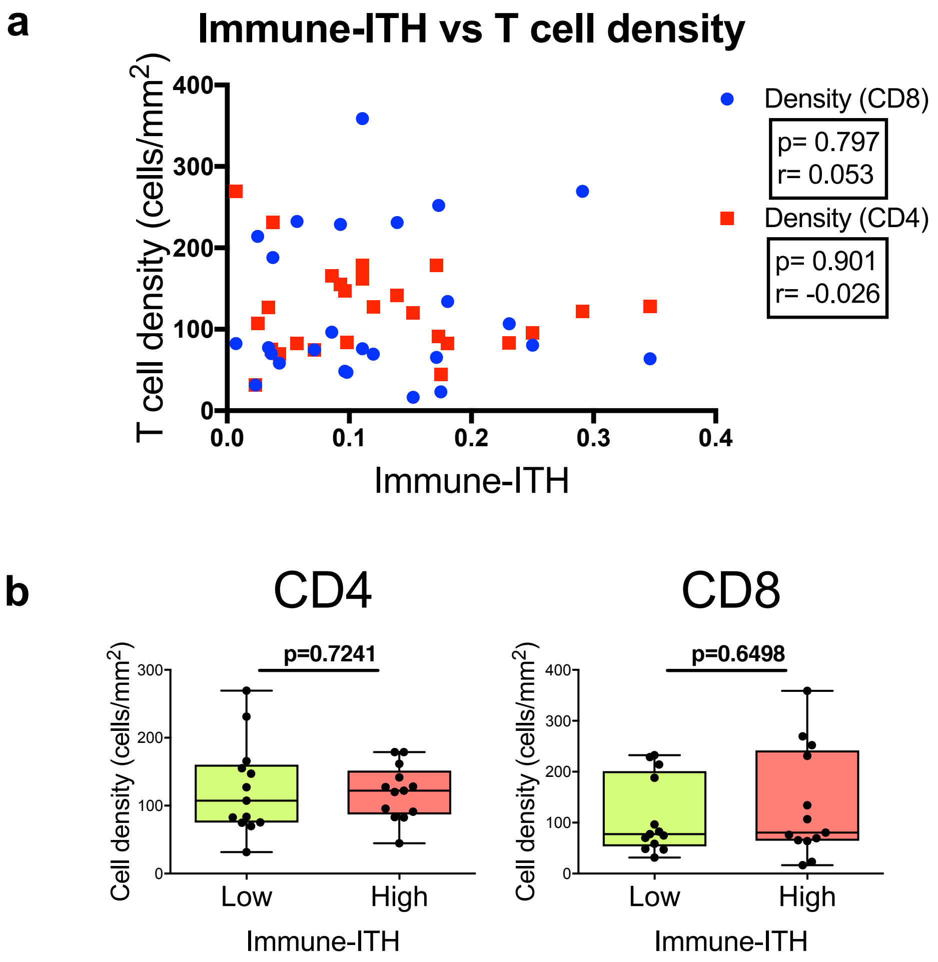

Supplementary Fig. 7: Relationship between immune-ITH and tissue T cell densities.
a CD4 or CD8 tumour T cell densities were correlated with immune-ITH from each patient. Spearman's r and p values were indicated.
b CD4 or CD8 tissue T cell densities in tumours with low or high immune–ITH. Data was shown by box plot. The whiskers represent minimum and maximum values, the band inside the box is the median and box edges show the first and third quartiles. P values are not significant (ns) by two-sided Mann-Whitney U-test.
a and b CD8 T cells and CD4 T cells densities were quantified from 10 regions from each tumour per patient (n=26 patients).

a

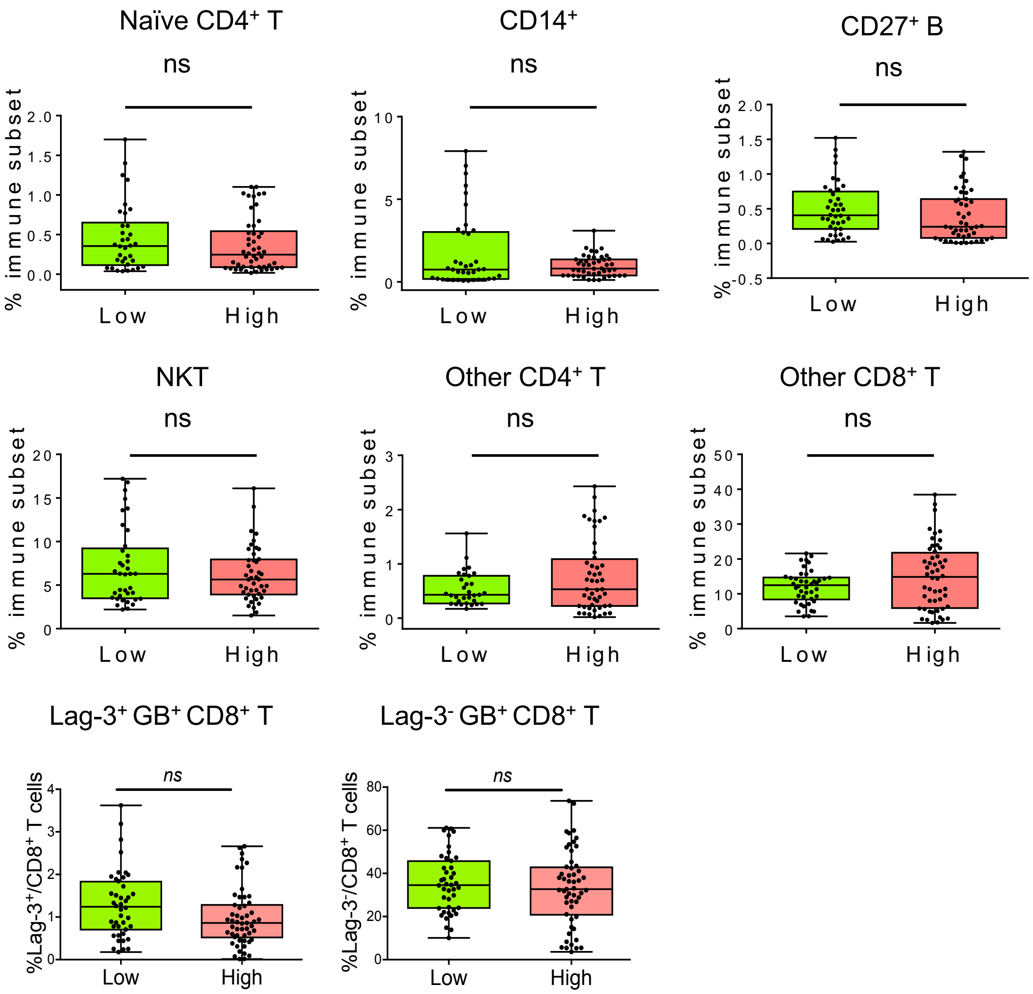

Supplementary Fig.8: Relationship of immune subsets with immune-ITH.
a Proportions of other immune subsets and Lag-3+ or – CD8+ T cells in tumours with low or high immune–ITH. Data was shown by box plot. The whiskers represent minimum and maximum values, the band inside the box is the median and box edges show the first and third quartiles. P values are either * p< 0.05 or not significant (ns) by two-sided Mann-Whitney U-test. n= 95 total tumour sectors.

a

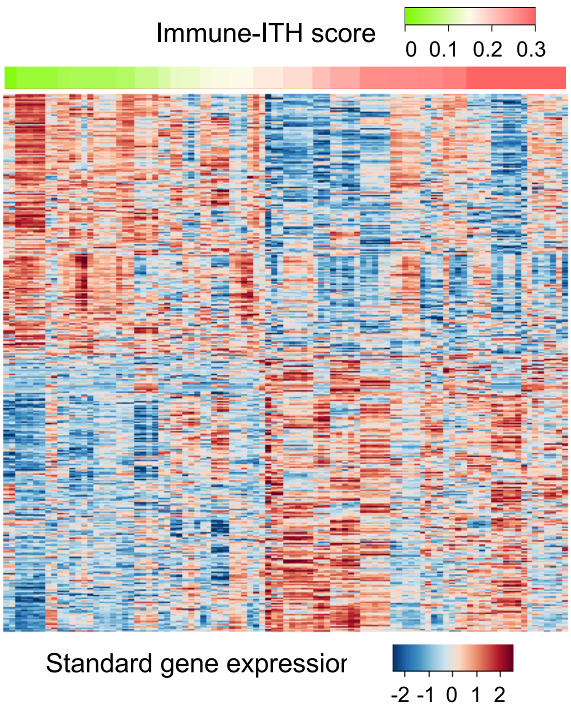

Supplementary Fig. 9: Transcriptomic signature of immune-ITH

a Heatmap showing relative expressions of 1,709 differentially expressed genes (DEGs) between tumours with low or high immune–ITH analyzed using R package limma v3.38.3 with False Discovery Rate (FDR)< 0.01 (two-sided) using Benjamini-Hochberg procedure accounted for multiple comparisons. Source data are provided as a Source Data file.

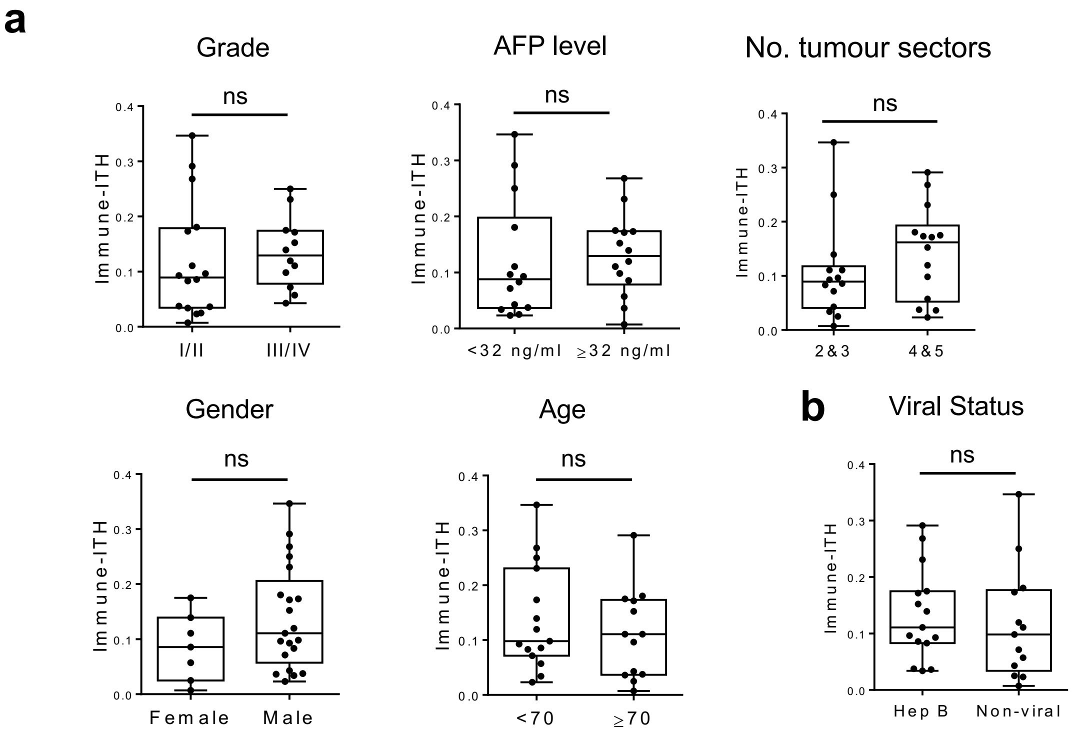

Supplementary Fig. 10: Association immune-intratumoural heterogeneity (immune-ITH) with various clinical parameters.

a Immune–ITH in tumours with grade I/II versus III/IV; < 32 ng/ml versus ≥ 32ng/ml alpha-fetoprotein (AFP) level (cut-off at median level = 32 ng/ml); female or male patients; < 70 versus ≥ 70 years old (cut-off at median level = 70 years old) and tumours with 2 or 3 versus 4 or 5 number of sectors.

b Immune–ITH in tumours with Hepatitis B (HBV)-related versus non-viral-related;

a and b The whiskers represent minimum and maximum values, the band inside the box is the median and box edges show the first and third quartiles. *P* values were calculated by two-sided Mann-Whitney U-test. Clinical data from n=28 patients.

a

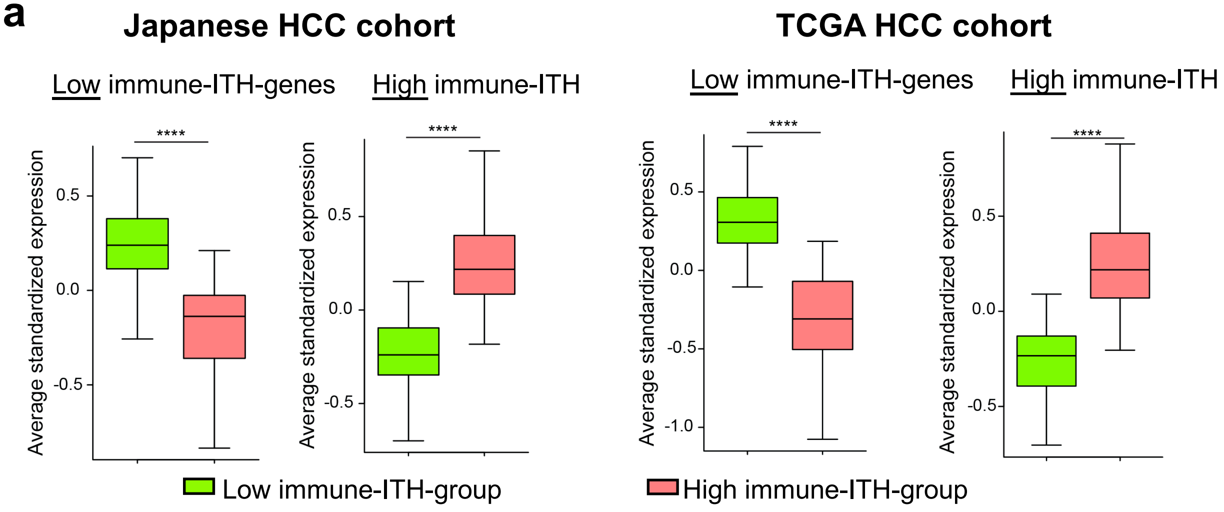

b

Immune-ITH Analysis	14 low vs 14 high	15 low (Add A004) vs 13 High	13 low vs 15 high (Add C005)
Robustness of target genes in public cohort (FDR < 0.01)	No. of genes: 1709/1709 (100%)	No. of genes (p-value): 1707/1709 (99.9%)	No. of genes (p-value): 1623/1709 (95%)
Public HCC cohort overall survival<br>1. Japanese:<br>2. TCGA	1. $P = 0.00025$<br>2. $P = 4.1 \times 10^{-5}$	1. $P = 8.3 \times 10^{-5}$<br>2. $P = 5.1 \times 10^{-6}$	1. $P = 0.0055$<br>2. $P = 0.0042$

c

% of differentially expressed genes		*P* values of survival profiles in Japanese cohort		*P* values of survival profiles in TCGA cohort	
Mean	Range	Mean	Range	Mean	Range
99.80%	[95.7% - 100%]	0.00068	[4.9×10^{-5} - 4.8×10^{-3}]	0.000041	[1.8×10^{-7} - 3.0×10^{-4}]

Supplementary Fig. 11: The analysis of HCC public cohorts with low versus high immune-intratumoural heterogeneity (ITH).

a Expression of genes associated for either low or high immune-ITH in low (green) vs high (red)-immune-ITH groups in two public cohorts. Two-tailed P values were based on unpaired t-test, **** p< 0.0001. The whiskers represent 5th and 95th percentiles, the band inside the box is the median and box edges show the first and third quartiles. Japanese cohort (n=203) and TCGA cohort (n=315).

b Robustness test by shifting one patient from high- to low- or from low- to high-immune-ITH groups. Over 95% of the target genes remained significant and still capable of segregating patient overall survival (FDR < 0.01). Two-tailed P values were calculated from Kaplan-Meier analysis with log-rank test.

c Robustness test by leave one (patient) out analysis. Each patient was removed once from the original data. Over 95% of the target genes remained significant and still capable of segregating patient overall survival (FDR < 0.01). Mean and range of percentages of target genes that remained significant in each removal and their corresponding two-tailed P values by log-rank test of survival profiles in Japanese and TCGA cohorts were shown.
