# Supplementary material for: Intratumoural immune heterogeneity as a hallmark of tumour evolution and progression in hepatocellular carcinoma
Source: Nat Commun. 2021 Jan 11;12:227. doi: 10.1038/s41467-020-20171-7 (PMC7801667; doi:10.1038/s41467-020-20171-7)
Supplement: Supplementary file 3 — Reporting Summary [file 41467_2020_20171_MOESM3_ESM.pdf]

nature research

Corresponding author(s): Valerie Chew, Weiwei Zhai and Pierce Chow

Last updated by author(s): Nov 12, 2020

Reporting Summary

Nature Research wishes to improve the reproducibility of the work that we publish. This form provides structure for consistency and transparency in reporting. For further information on Nature Research policies, see our Editorial Policies and the Editorial Policy Checklist.

Statistics

For all statistical analyses, confirm that the following items are present in the figure legend, table legend, main text, or Methods section.

n/a	Confirmed	
☐	☒	The exact sample size (n) for each experimental group/condition, given as a discrete number and unit of measurement
☐	☒	A statement on whether measurements were taken from distinct samples or whether the same sample was measured repeatedly
☐	☒	The statistical test(s) used AND whether they are one- or two-sided<br>*Only common tests should be described solely by name; describe more complex techniques in the Methods section.*
☐	☒	A description of all covariates tested
☐	☒	A description of any assumptions or corrections, such as tests of normality and adjustment for multiple comparisons
☐	☒	A full description of the statistical parameters including central tendency (e.g. means) or other basic estimates (e.g. regression coefficient) AND variation (e.g. standard deviation) or associated estimates of uncertainty (e.g. confidence intervals)
☐	☒	For null hypothesis testing, the test statistic (e.g. F, t, r) with confidence intervals, effect sizes, degrees of freedom and P value noted<br>*Give P values as exact values whenever suitable.*
☒	☐	For Bayesian analysis, information on the choice of priors and Markov chain Monte Carlo settings
☒	☐	For hierarchical and complex designs, identification of the appropriate level for tests and full reporting of outcomes
☐	☒	Estimates of effect sizes (e.g. Cohen's d, Pearson's r), indicating how they were calculated

Our web collection on statistics for biologists contains articles on many of the points above.

Software and code

Policy information about availability of computer code

Data collection	Cytometry by Time of Flight (CyTOF) data was collected using the CyTOF® 6.7 system control software (Fluidigm) came equipped with Helios CyTOF machine. RNA sequencing and whole genome sequencing perform at Genome Institute Singapore (GIS).
Data analysis	All softwares used for data analysis were listed in the methods section with citations. They include: EPIC with browser-based R Shiny app 'SciAtlasMiner' (Yeo et al. Nat Biotechnol 2020), Phenograph (v.1.5.2), fast interpolation based t-distributed neighbour embedding (fi-tSNE, v.1.0.1), FlowJo v.10.2, R v3.4.4, STAR v2.5.2a , RSEM v1.3.0 , DEseq2 v1.22.2, ggplot2 v3.1.1, limma v3.38.3, CIBERSORT (Newman et al. Nat Methods 2015), Burrows–Wheeler Aligner v0.7.12, Genome Analysis Tool Kit v3.1, neighbor-joining algorithm (Saitou et al. MolBev 1987), Sequenza v2.1.2, LOHHLA (McGranahan N, et al. Cell 2017), Mutect v.1.1.7, personalized Variant Antigens by Cancer Sequencing (pVacSeq) v4.0.10, VEP v86, Polysolver v1.0, GISTIC v 2.0, DAVID v6.8, and GraphPad Prism v.7

For manuscripts utilizing custom algorithms or software that are central to the research but not yet described in published literature, software must be made available to editors and reviewers. We strongly encourage code deposition in a community repository (e.g. GitHub). See the Nature Research guidelines for submitting code & software for further information.

Data

Policy information about availability of data

All manuscripts must include a data availability statement. This statement should provide the following information, where applicable:

- Accession codes, unique identifiers, or web links for publicly available datasets
- A list of figures that have associated raw data
- A description of any restrictions on data availability

The DNA and RNA sequencing data generated in this study are deposited in European Genome-phenome Archive (EGA), which is hosted by The European Bioinformatics Institute (EBI) under the accession code: EGAS00001003814 [https://www.ebi.ac.uk/ega/studies/EGAS00001003814]. The remaining data are

available within the Article, Supplementary Information and Source data provided with this paper.

Field-specific reporting

Please select the one below that is the best fit for your research. If you are not sure, read the appropriate sections before making your selection.

☒ Life sciences ☐ Behavioural & social sciences ☐ Ecological, evolutionary & environmental sciences

For a reference copy of the document with all sections, see nature.com/documents/nr-reporting-summary-flat.pdf

Life sciences study design

All studies must disclose on these points even when the disclosure is negative.

Sample size	We included all patients from a prospective cohort of HCC patients who underwent resection as first line therapy with followup until recurrence. No statistical methods were used to predetermine sample size. All patients tumour regions with sufficient quality of DNA/RNA/CyTOF data were included.
Data exclusions	No data was excluded
Replication	No replication. Experiment was performed on all samples with single set of data produced from the limited biological sample available.
Randomization	This is not relevant to this study as it is not an interventional study. Samples were grouped according to immune heterogeneity.
Blinding	This is not relevant to this study as no treatment is involved. The identity of the patients was blinded to the team collecting and analyzing the data.

Reporting for specific materials, systems and methods

We require information from authors about some types of materials, experimental systems and methods used in many studies. Here, indicate whether each material, system or method listed is relevant to your study. If you are not sure if a list item applies to your research, read the appropriate section before selecting a response.

Materials & experimental systems

n/a	Involved in the study	
☐	☒	Antibodies
☒	☐	Eukaryotic cell lines
☒	☐	Palaeontology and archaeology
☒	☐	Animals and other organisms
☐	☒	Human research participants
☐	☒	Clinical data
☒	☐	Dual use research of concern

Methods

n/a	Involved in the study	
☒	☐	ChIP-seq
☐	☒	Flow cytometry
☒	☐	MRI-based neuroimaging

Antibodies

Antibodies used	For CyTOF, we used metals-tagged antibodies either preconjugated or conjugated in-house according to the manufacturer's instructions (Fluidigm). The catalogue numbers and working dilutions were stated in supplementary Table 2. For immunohistochemistry staining, we used anti-human CD4 (Abcam, clone EPR6855, 1:200), CD8 (DAKO, clone C8/144B, 1:200) and Foxp3 (Abcam, clone 236A/E7, 1:100) antibodies.
Validation	Each primary antibodies purchased was validated for the species and application with information available from the manufacturer's website. Prior to this study, we have also tested all these antibodies on various immune cells taken from healthy individuals and cancer patients and published several articles, which include: Chew et al PNAS 2017, Chew et al. Gut 2018 and Lim and Lee et al. Gut 2018.

Human research participants

Policy information about studies involving human research participants

Population characteristics	Please refer to clinical information of our HCC patient cohort in Supplementary Table 1.<br><br>Patients were recruited into study according to the following eligibility criteria (taken from the study protocol).<br>Inclusion criteria:<br>1. Male and female patients, 21 to 90 years of age at the time of signature of the informed consent form.<br>2. Clinically AND histologically proven HCC or hepatocellular-cholangiocarcinoma after liver resection. Clinically diagnosed HCC or hepatocellular-cholangiocarcinoma that turns out to be peripheral cholangiocarcinoma on histology can be included.<br>3. HCC or hepatocellular-cholangiocarcinoma limited to the liver with no extra-hepatic metastasis on CT or MRI of the abdomen and chest (defined as lymph node <2 cm, lung modules < 1 cm, further lymph nodes < 2 cm) according to the AASLD criteria.<br>4. R0 or R1 resection on histology.<br>5. Eligibility according to tumour size based on pre-op imaging:<br>a. Large tumour >= 5 cm (preferred)<br>b. Smaller tumours >=2cm and < 5cm<br>c. Multifocal tumours - maximum of 3 total tumours with at least one with size >=2cm detected from CT-scan.<br>6. Child-Pugh ≤ 7 points without clinical ascites before surgery.<br>7. ECOG performance status 0-1 before surgery.<br>8. The patient has received no anti-cancer specific treatment for HCC or hepatocellular-cholangiocarcinoma eg. previous liver resection, loco-regional therapy (e.g. RFA, TACE, SIRT), radiotherapy, immunotherapy, chemotherapy or neo-adjuvant chemotherapy other than the planned surgery. However, patient who has received previous HCC resection more than 5 years ago is deemed to have a de-novo liver tumour and therefore can be included.<br>10. Adequate bone-marrow reserve, renal function and hepatic function as assessed by standard laboratory criteria<br><br>Exclusion Criteria<br>1. Patients unable to give inform consent.<br>2. Single lesion < 2 cm at the time of pre-op imaging.<br>3. The patient has previous or concomitant malignancies at other sites, except effectively treated non-melanoma skin cancers or carcinoma in situ of the cervix or effectively treated malignancy that has been in remission for over 5 years and highly likely to have been cured.<br>4. Encephalopathy<br>5. The patient has received a major organ allograft.<br>6. The patient is known to be positive for the Human Immunodeficiency Virus (HIV).<br>7. The patient has an uncontrolled bleeding disorder.<br>8. The patient has uncontrolled congestive heart failure or hypertension, unstable heart disease (coronary artery disease or myocardial infarction) or uncontrolled arrhythmia at the time of enrolment.<br>9. The patient has psychiatric or addictive disorders that may compromise his/her ability to give informed consent, or to comply with the study procedures.<br>10. The patient has other concurrent severe medical problems, unrelated to the malignancy, that would significantly limit full compliance with the study or expose thse patient to unacceptable risk.<br>11. The patient has received any investigational or non-registered medicinal product (drug or vaccine) within the 30 days preceding the date of enrolment, or plans to receive such a drug during the study period.<br>12. For female patients: the patient is pregnant or lactating.<br><br>Ineligibility following screening<br>1. Insufficient or poor quality DNA/RNA for genome analysis at baseline.<br>2. Insufficient cells for immunology analysis at baseline.<br>3. Clinically diagnosed HCC or hepatocellular-cholangiocarcinoma that turns out to be non-HCC.<br>4. The patient is unable to comply with protocol requirements.
Recruitment	Patients who were scheduled for resection were pre-screened for eligibility to join this study. All patients were assigned a study ID such that the patients could not identify themselves in study publications. All human samples, tissue and blood, were linked to the study ID such that they were de-identified and tracked on a centralised database overseen by the study sponsor only.<br>Informed consent for entry into the the study was mandatory and obtained from every patient.
Ethics oversight	The study was approved by the Central Institution Review Board (CIRB) of SingHealth of which all National Cancer Center Singapore, Singapore General Hospital and National University Hospital were constituent members (CIRB Ref: 2016/2626 and 2018/2112). Each patient gave informed written consent.

Note that full information on the approval of the study protocol must also be provided in the manuscript.

Clinical data

Policy information about clinical studies

All manuscripts should comply with the ICMJE guidelines for publication of clinical research and a completed CONSORT checklist must be included with all submissions.

Clinical trial registration	https://clinicaltrials.gov/ct2/show/NCT03267641
Study protocol	This is a prospective, observational cohort study following standard of care- liver cancer resection and routine follow-up with primary outcome measured as time to recurrence. Study protocol is approved by CIRB upon patient consent to collect pre surgical blood and post surgical tissues.
Data collection	Routine follow-up. No post resection treatment until recurrence.
Outcomes	Time to recurrence (observational)

Flow Cytometry

Plots

Confirm that:

- [x] The axis labels state the marker and fluorochrome used (e.g. CD4-FITC).
- [x] The axis scales are clearly visible. Include numbers along axes only for bottom left plot of group (a 'group' is an analysis of identical markers).
- [x] All plots are contour plots with outliers or pseudocolor plots.
- [x] A numerical value for number of cells or percentage (with statistics) is provided.

Methodology

Sample preparation	Single cell suspensions were obtained from surgically resected multi-region HCC tumors and adjacent non-tumour tissue by enzymatic digestion as previously described in Chew et al. PNAS 2017. PBMC from blood were obtained Ficoll-Paque PLUS (GE Healthcare, UK) density centrifugation as per manufacturer instructions. All samples were cryopreserved until the point of use.
Instrument	Helios mass cytometer (Fluidigm, USA)
Software	Data was collected with CyTOF® 6.7 system control software (Fluidigm) came equipped with Helios CyTOF machine. Data analysis were performed using EPIC (Yeo et al. Nat Biotechnol 2020) and FlowJo v.10.2
Cell population abundance	All cells were thawed and analyzed with CyTOF without prior sorting.
Gating strategy	Detailed gating strategy for key cell subsets was provided in supplementary fig. 3a

- [x] Tick this box to confirm that a figure exemplifying the gating strategy is provided in the Supplementary Information.
